# Explainable Clinical Decision Support for Metabolic Index Prediction in Gout Patients Using GA-Optimized Ensemble Learning Models

**DOI:** 10.3390/diagnostics16152418

**Published:** 2026-07-31

**Authors:** Fatih Bal, Osman Cüre

**Affiliations:** 1 Department of Software Engineering, Faculty of Engineering, Kirklareli University, Kirklareli 39010, Turkey; fatihbal@klu.edu.tr; 2 Department of Internal Medicine, Division of Rheumatology, Recep Tayyip Erdoğan University, Rize 53200, Turkey

**Keywords:** gout, insulin resistance, ensemble learning, genetic algorithm, metabolic index prediction, SHAP

## Abstract

**Background/Objectives**: Metabolic indices such as HOMA-IR, METS-IR and TyG play a critical role in determining cardiometabolic risk and insulin resistance in clinical practice. This study aims to simultaneously predict these three indices using ensemble learning models, based on demographic, clinical, and laboratory data from patients with gout. **Methods**: Demographic, retrospective clinical, and laboratory data from 411 patients diagnosed with gout were analyzed, and a logarithmic transformation was applied to address skewness in the target variable. A genetic algorithm was used to identify relevant features for predicting metabolic indices, and four scenarios were designed. Five ensemble learning models were optimized using fine-tuning with Optuna. The models’ decisions were clinically validated using SHAP analysis. **Results**: Across all scenarios, the CatBoost model combined with the GA + Log-Transformed framework achieved the highest prediction accuracy and the lowest error rates. Notably, when predicting the TyG index, it simulated the underlying formula with near-perfect accuracy. SHAP analysis confirmed that CatBoost successfully mapped clinical pathophysiology by prioritizing insulin levels for HOMA-IR, BMI for METS-IR, and triglyceride variance for the TyG index. **Conclusions**: The proposed GA + Log-Transformed CatBoost workflow provides a highly accurate, non-invasive and interpretable clinical decision support system. This study demonstrates that the ensemble architecture effectively captures complex pathophysiological patterns aligned with medical knowledge rather than merely memorizing statistical noise.

## 1. Introduction

Gout is a chronic inflammatory rheumatic disease caused by the accumulation of monosodium urate crystals in the joints and periarticular tissues. The condition is not limited to hyperuricaemia alone; it is closely associated with metabolic syndrome, insulin resistance, obesity, chronic kidney disease (CKD), hypertension and cardiovascular disease [1,2]. Studies conducted in recent years, in particular, have shown that gout is not merely a joint disease but a complex inflammatory process associated with systemic metabolic disorders. Consequently, the assessment of metabolic status in patients with gout has become an important clinical approach in predicting disease activity, prognosis and the risk of complications [3,4]. Insulin resistance is a key mechanism in the pathogenesis of gout [5]. Insulin resistance can increase serum uric acid levels by reducing renal urate excretion, can exacerbate the inflammatory response, and, in conjunction with components of metabolic syndrome, may contribute to a more aggressive course of the disease [6]. For this reason, metabolic indices used to assess insulin resistance are increasingly being employed in clinical practice. Parameters such as the Homeostasis Model Assessment of Insulin Resistance (HOMA-IR), the Triglyceride-Glucose (TyG) Index, the Triglyceride/HDL ratio (TG/HDL) and the Metabolic Score for Insulin Resistance (METS-IR) are recognised as robust biomarkers for assessing both metabolic burden and cardiometabolic risk [7,8,9]. Although HOMA-IR is a classic indicator of insulin resistance based on fasting glucose and insulin levels, the fact that insulin measurements cannot be routinely performed at every centre may limit its use [10]. In contrast, the TyG index stands out as a more practical and cost-effective alternative, as it is calculated from triglyceride and fasting glucose levels [11]. On the other hand, METS-IR provides a more comprehensive metabolic profile by assessing glucose, triglycerides, HDL and BMI together [7]. It is thought that these indices may be associated with the clinical course of gout, the frequency of attacks, the level of inflammation and the burden of comorbidity. Although there are studies in the literature on assessing metabolic indices in gout patients, few compare the relative performance of these indices in predicting disease progression and analyse them using advanced machine learning methods. Traditional statistical methods are often inadequate for capturing the complex, non-linear relationships between variables [12,13]. Machine learning methods offer significant advantages, particularly in multi-dimensional clinical datasets, for modelling the relationships between metabolic parameters and disease activity more accurately. Although there has been an increase in the number of applications using machine learning methods in studies on metabolic indices, there are significant gaps in the literature. Studies in this field have generally focused on predicting a single metabolic index and have addressed two classification problems related to insulin resistance using simplified feature sets. However, there is a lack of a unified computational framework that predicts multiple metabolic indices using clinical gout datasets. Furthermore, the interaction between tree-based gradient boosting models and the combination of feature selection via GA and logarithmic transformation to uncover pathophysiological mechanisms has not been sufficiently investigated. This study presents a comprehensive framework to address these gaps.

This study presents a comprehensive framework for predicting key indicators of insulin resistance—such as the HOMA-IR, METS-IR, and TyG indices—using ensemble learning models on a multidimensional clinical dataset from patients with gout, to address existing gaps in the literature. Its contributions to the literature are summarised under four main headings:

i. To establish a methodological standard by analysing metabolic indices associated with different mathematical formulas using a concurrent data processing pipeline.
ii. To apply a log transformation to the right-skewed target data and examine its effects on model performance from both methodological and clinical perspectives.
iii. To present a hybrid data engineering strategy by applying a Genetic Algorithm-based feature selection to shift the data space to the most optimal points.
iv. SHAP analysis was utilized to elucidate the feature importance underlying CatBoost decisions, thereby demonstrating how the model prioritizes clinical features that strongly align with known pathophysiological mechanisms.

By incorporating all four of these characteristics, this study plays a pioneering role in moving clinical decision support systems beyond the “black-box” stage and enhancing the reliability of medical predictions.

## 2. Related Works

In recent years, AI and ML models have demonstrated remarkable capabilities in processing and classifying medical images, signal processing, and risk classification in various clinical support systems. For example, quantum machine learning frameworks have achieved high accuracy in diagnostic tasks such as cardiac MRI analysis [14] and in identifying abnormalities like paediatric supraventricular tachycardia in ECG images using hybrid ensemble learning models [15]. Furthermore, advanced CNN and ViT models are yielding highly successful results in MRI and X-ray images. In line with developments in this field, ML approaches are increasingly being used to predict metabolic indices.

In this section of the study, we reviewed the literature on studies that estimated the HOMA-IR, METS-IR, and TyG indices. In a study, researchers [16] used machine learning models to classify insulin resistance in non-diabetic individuals from only fasting blood glucose levels and easily measured physical data. In their research, machine learning models were trained on data from fasting blood glucose, age, gender, ethnicity, height, weight, Body Mass Index (BMI), waist circumference, pulse, and blood pressure to classify HOMA-IR, METS-IR, and TyG indices. Threshold values of 2.5 for HOMA-IR, 8.85 for TyG, and 41.33 for METS-IR were determined for classification. In the classification analyses, the model yielding the best results was CATBOOST for the HOMA-IR and TyG indices, whilst TabKANet was the best for METS-IR. In another study, the performance of a range of machine learning models was comprehensively evaluated to predict insulin resistance in Korean adults [17]. A dataset comprising 1411 features—including demographic, anthropometric, biochemical, genetic, and nutritional parameters—from 8842 patients aged 40–74 was used to predict insulin resistance. From the 1411 features, 99 features associated with insulin resistance were manually selected by the researchers. In this scenario with 99 features, the model that yielded the highest result among the seven models was XGB (86.80%). In the 15-feature scenario, the highest result was again achieved by the XGB model (87.70%). When the number of features was reduced to 9, the ANN achieved the highest performance (86.20%). Furthermore, SHAP analysis was utilised to interpret the positive and negative effects of the models evaluated for performance. In a separate study, researchers [18] analysed the performance of artificial intelligence models to clinically predict liver stiffness in individuals across the USA. In their study, they discussed the effects of blood tests, general patient characteristics, and indicators of insulin resistance on model performance. In particular, the inclusion of METS-IR index indicators in the dataset improved the model’s ability to predict liver stiffness. The dataset they used comprised data from 3564 patients, including general demographic information, physical examination findings, laboratory blood test results, and target variables. In their study, they evaluated the performance of the XGB model across three scenarios. To assess model accuracy and performance, they demonstrated agreement across performance metrics using a Bland–Altman plot. In a study aimed at detecting insulin resistance in children and adolescents at an early stage, the researchers [19] utilised a dataset comprising physical examination data, laboratory results and dietary habits. The dataset contains 41 features and 827 data points. In this study, which aimed to predict the HOMA-IR index, univariate logistic regression (UVLR) analysis was used for feature selection. Scores of 3.0 or higher in the HOMA-IR data were considered indicative of insulin resistance. In the study, which utilised a range of machine learning models, the Support Vector Machine (SVM) model yielded the highest results. In another study, researchers [20] used machine learning approaches to detect insulin resistance at an early stage in children aged 6–12 and analysed their performance. The dataset, compiled from demographic data, daily habits and laboratory blood tests, contains 17 features and 503 data points. Unlike other studies, they used the SMOTE technique—a synthetic data augmentation method—to balance the data between healthy children and those with insulin resistance. The researchers used the Recursive Feature Elimination (RFE) technique to identify the most significant risk factors and examined the performance of five different machine learning models to compare the results. The model that yielded the best results was XGB. In another study, a high-accuracy machine learning model for predicting insulin resistance in individuals without diabetes was introduced. The researchers [21] used a dataset comprising 9 key features and a total of 26.737 records (NHANES = 14,211, MJ = 12,526) sourced from the USA and Taiwan. In the study, which applied SMOTE to address class imbalance, the performance of four models was analysed. Additionally, they conducted a SHAP analysis to explain which features the models prioritised when predicting HOMA-IR. The model yielding the best results was XGB. Finally, in a separate study, researchers [22] compared the performance of the HOMA-IR and TyG indices in detecting metabolic syndrome. The study utilised a dataset obtained from the Korean Genome and Epidemiology longitudinal prospective cohort. Although data were available for 9333 participants, after applying exclusion criteria, data from 3580 adults were analysed. During the feature and data filtering stage, Single Nucleotide Polymorphisms (SNPs) were used to identify genetic variants, and the SNPs were ranked by their logistic regression *p*-values. Of these ranked data, 5 SNPs with strong statistical associations were used to estimate the model. The combination model using the 5 SNPs alongside the TyG index yielded the best results, whilst traditional biostatistical methods were employed for estimation.

A review of the existing literature reveals that machine learning approaches have primarily focused on predicting a single metabolic index, with an emphasis on classification. This study is the first to simultaneously evaluate three distinct metabolic indices—each with its own mathematical formula—for determining insulin resistance, using a single data-processing pipeline. Furthermore, it presents a hybrid approach that combines feature selection using a genetic algorithm—rather than traditional biostatistical methods—with logarithmic transformation. SHAP analysis was applied to methodologically explain the clinical causal relationship in artificial intelligence.

## 3. Materials and Methods

### 3.1. Dataset Source and Clinical Indicators

**Figure 1:**
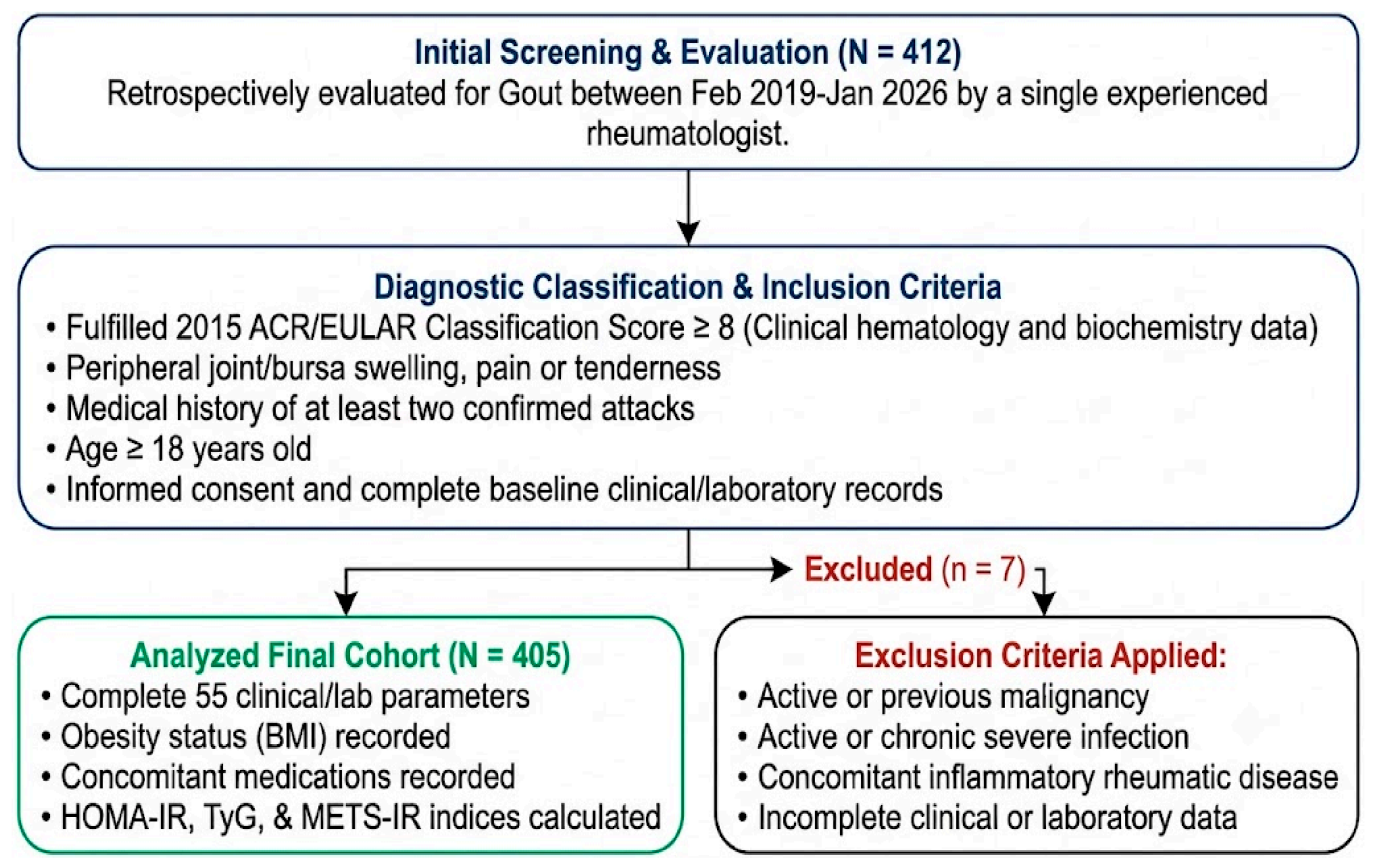
Flowchart of the patient selection process, diagnostic classification criteria (2015 ACR/EULAR score ≥ 8), inclusion/exclusion criteria, and final analyzed analytical study population.

The dataset used in the study was obtained from the Department of Rheumatology at Rize Recep Tayyip Erdoğan University, Rize Training and Research Hospital, in accordance with ethical guidelines, and approval was obtained from the ethics committee. The dataset contains clinical data from 412 patients. Patients with gout were classified according to the 2015 American College of Rheumatology/European League Against Rheumatism (ACR/EULAR) classification criteria [23]. The entry criterion required at least one episode of swelling, pain, or tenderness in a peripheral joint or bursa. Subsequently, clinical and laboratory items were scored, including the pattern of joint involvement (ankle/midfoot: 1 point; first metatarsophalangeal joint: 2 points), characteristics of symptomatic episodes (erythema, inability to tolerate touch or pressure, and difficulty walking; 1–3 points), time course of episodes (one typical episode: 1 point; recurrent typical episodes: 2 points), presence of a clinical tophus (4 points), and serum urate level (<4 mg/dL: −4 points; 6–<8 mg/dL: 2 points; 8–<10 mg/dL: 3 points; ≥10 mg/dL: 4 points). Conventional radiographs were evaluated for gout-related structural damage (erosions) according to the ACR/EULAR classification criteria, whereas ultrasonography, dual-energy computed tomography (DECT), and synovial fluid monosodium urate crystal analysis were not routinely performed. Patients with a total score of ≥8 were classified as having gout. All patients were evaluated, and the ACR/EULAR classification criteria were applied by a single experienced rheumatologist. Although synovial fluid crystal analysis was not available in all cases, every patient met the relevant classification criteria, and their diagnoses of gout were confirmed by a rheumatologist experienced in the field, based on clinical, laboratory and imaging findings. In addition, all patients had a history of at least two gout attacks, as confirmed in their medical records. Data were retrospectively collected from the Hospital Information Management System (Akgun Software, Trabzon, Turkey, v26.3.238) for patients who presented for routine check-ups between February 2019 and January 2026. Inclusion criteria were age 18 years or older, written informed consent, and complete clinical and laboratory data. Exclusion criteria included pregnancy, a history of current or previous malignancy, active or chronic infection, and the presence of concomitant inflammatory rheumatological disease. Furthermore, individuals with incomplete clinical and/or laboratory data were excluded from the study. The demographic distribution of the patients is shown in Table 1. The raw dataset contains 55 distinct features designed to reflect patients’ clinical and metabolic statuses. The HOMA-IR, TyG Index, and METS-IR indices, which are associated with these features, were identified as key indicators. In addition to all this, factors affecting insulin resistance and metabolic profiles—namely obesity status and medication use—were meticulously recorded and added to the feature space in the dataset. Obesity was measured using BMI. Antidiabetic medicines (glucose-lowering therapy), lipid-lowering and uric acid-lowering medicines (febuxostat, colchicine) and cardiovascular medicines (ACE inhibitors/ARBs, beta-blockers, calcium channel blockers and diuretics) were recorded in the dataset as distinct categorical variables. The detailed patient screening, diagnostic verification based on the 2015 ACR/EULAR classification criteria and eligibility filtering steps are summarized in the study flow chart in Figure 1.

### 3.2. Dataset Analysis

The distribution of demographic, clinical, and laboratory parameters in the dataset to be used in this study, by patients’ gender, is shown in detail in Table 1. Taking the distribution of the data into account, the Mann–Whitney-U (MWU) test was used for comparisons between groups, whilst the Interquartile Range (IQR) was used as a measure of central tendency and dispersion. Looking at the results in Table 2, the data for male and female patients do not resemble one another in clinical history and laboratory results, and they appear to exhibit two distinct profiles. The median age of female patients is 74.00, and the age at the first attack of gout is 67.50.

This is considerably higher than in male patients. When compared with clinical findings, this indicates that women experience gout attacks at an advanced age, following the postmenopausal period, which is consistent with the literature. Similarly, the BMI was higher in women than in men, whilst smoking and alcohol consumption were much more prevalent in men. This demonstrates that risk factors differ according to gender. Cigarette and alcohol consumption are lifestyle factors and were defined as categorical variables. Those who smoked more than five cigarettes a day for at least one year were classified as active smokers. Those who consumed alcohol more than once a week were classified as drinkers. In terms of laboratory findings, haemoglobin (Hgb), estimated glomerular filtration rate (eGFR), and alanine aminotransferase (ALT) levels were higher in men, whilst in women, erythrocyte sedimentation rate (ESR) and C-reactive protein (CRP) levels indicated that physiological differences and disease activity vary between the sexes. Taken together with these findings, the similar distributions of uric acid levels and disease duration across both gender groups represent a common feature of the disease’s chronic course.

Within the scope of the study, the fact that variables such as LDL, triglycerides, uric acid and WBC in the dataset did not show a statistically significant difference between the sexes (*p* > 0.05) and, consequently, the exclusion of these variables from the regression models was not accepted as a criterion; the reasons for this rejection were determined as follows:

i. The absence of a significant gender difference in a particular MWU test variable does not negate that variable’s predictive power for the target variable. When the Uric Acid variable is assessed, it may serve as a predictor of disease course and duration, even though it is similar between men and women. In the literature, it is suggested that, although serum uric acid levels are similar in women and men, the Uric Acid variable may provide prognostic information regarding the age of onset, clinical course and duration of exposure to hyperuricaemia. In particular, it has been reported that prolonged exposure to hyperuricaemia plays a decisive role in the development of gout and the progression of the disease [24].
ii. Statistically eliminating parameters whose clinical significance has already been established in the literature, based solely on their *p*-values, may cause the model to deviate from medical reality. This may lead to omitted-variable bias.
iii. Regression models capture complex relationships between variables in a dataset. A variable that appears insignificant may significantly improve the model’s performance when combined with another variable.

### 3.3. Dataset Preprocessing

**Figure 2:**
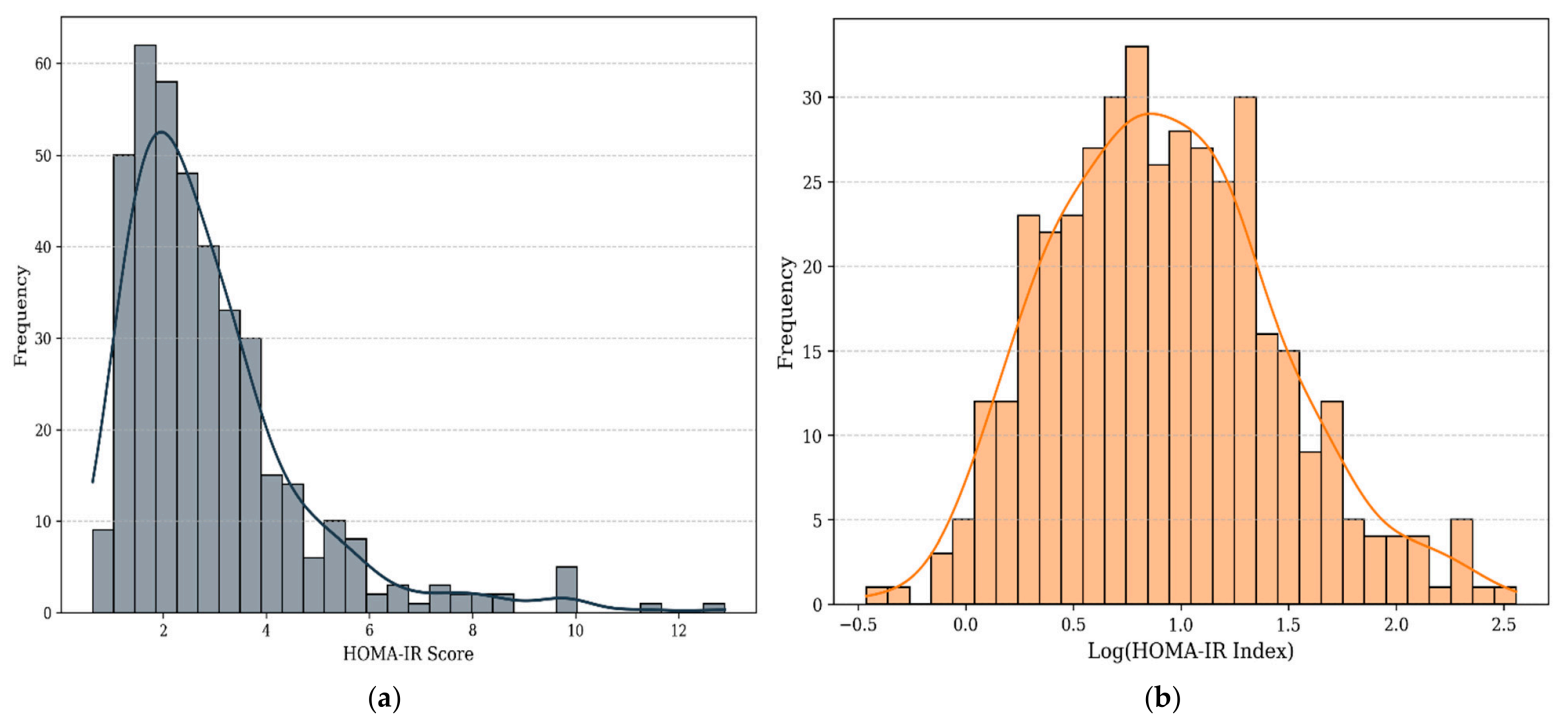
Distribution of HOMA-IR values: (**a**) Raw Data; (**b**) After applying the Log-Transformed framework.

This section of the study covers the stages involved in preparing the data for training. The general statistical assumption is that the dependent variable, denoted by the capital letter Y, must exhibit a homogeneous distribution across the independent variable(s), denoted by the capital letter X. Consequently, if the actual observations obtained from an experiment do not conform to a normal distribution, a log transformation can be applied to make the sample mean more consistent with a normal distribution [25]. Figure 2a shows the raw HOMA-IR values, and it is evident that the data exhibit a marked positive skew. Whilst most observations cluster between 1 and 4, values to the right are visible and considered outliers. This pattern is commonly observed in metabolic parameters. Whilst the observations for many patients may fall within the mild or normal ranges, a few patients may have high values. In Figure 2b, the right tail shown in Figure 2a has been smoothed, transforming the data distribution into a bell-shaped curve. The application of a logarithmic transformation in the analysis of biological markers is a standard procedure established in the literature [26,27,28,29]. The normality of all target variables before and after logarithmic transformation was assessed using the Shapiro–Wilk test and visual inspection of Q-Q plots. Since the models were trained to predict log(Y), the interpretations of the SHAP values reflect the feature contributions to the logarithmically transformed scale. As the raw distribution of HOMA-IR values was right-skewed, a logarithmic transformation was applied to improve model training performance. Following the transformation, the data’s conformity to a normal distribution was verified and is shown in Figure 2b.

**Figure 3:**
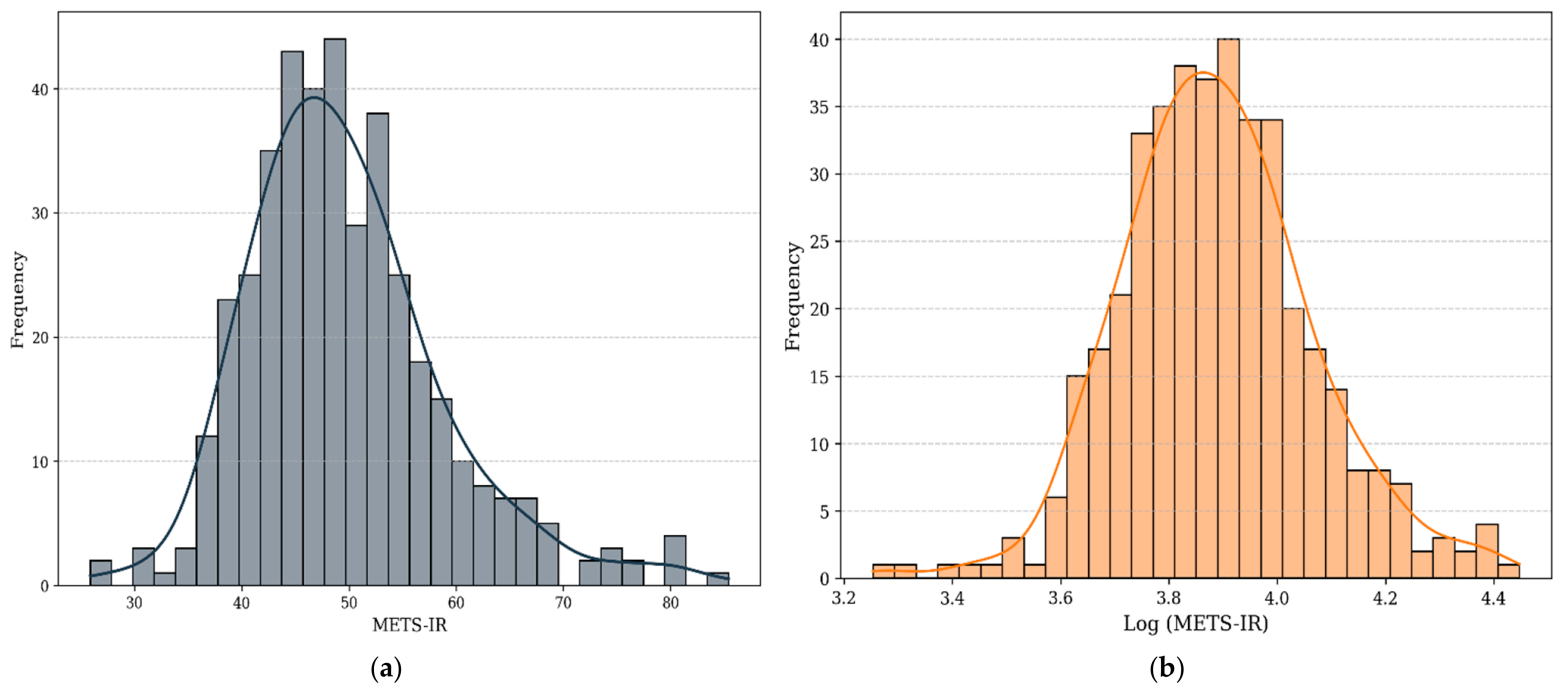
Distribution of METS-IR values: (**a**) Raw Data; (**b**) After applying the Log-Transformed framework.

As the METS-IR index is a composite score combining variables such as BMI, triglycerides, and fasting blood glucose, the raw data generally exhibit skewness. In Figure 3a, the data are right-skewed, with METS-IR values more concentrated in the 45–50 age group. Extreme cases among patients with gout are the primary cause of this skewness, and the arithmetic mean may be significantly influenced by these outliers. To minimise the impact of these outliers, a logarithmic transformation was applied to the METS-IR values, resulting in a symmetrical, bell-shaped distribution. The effect of this transformation is shown in Figure 3b.

**Figure 4:**
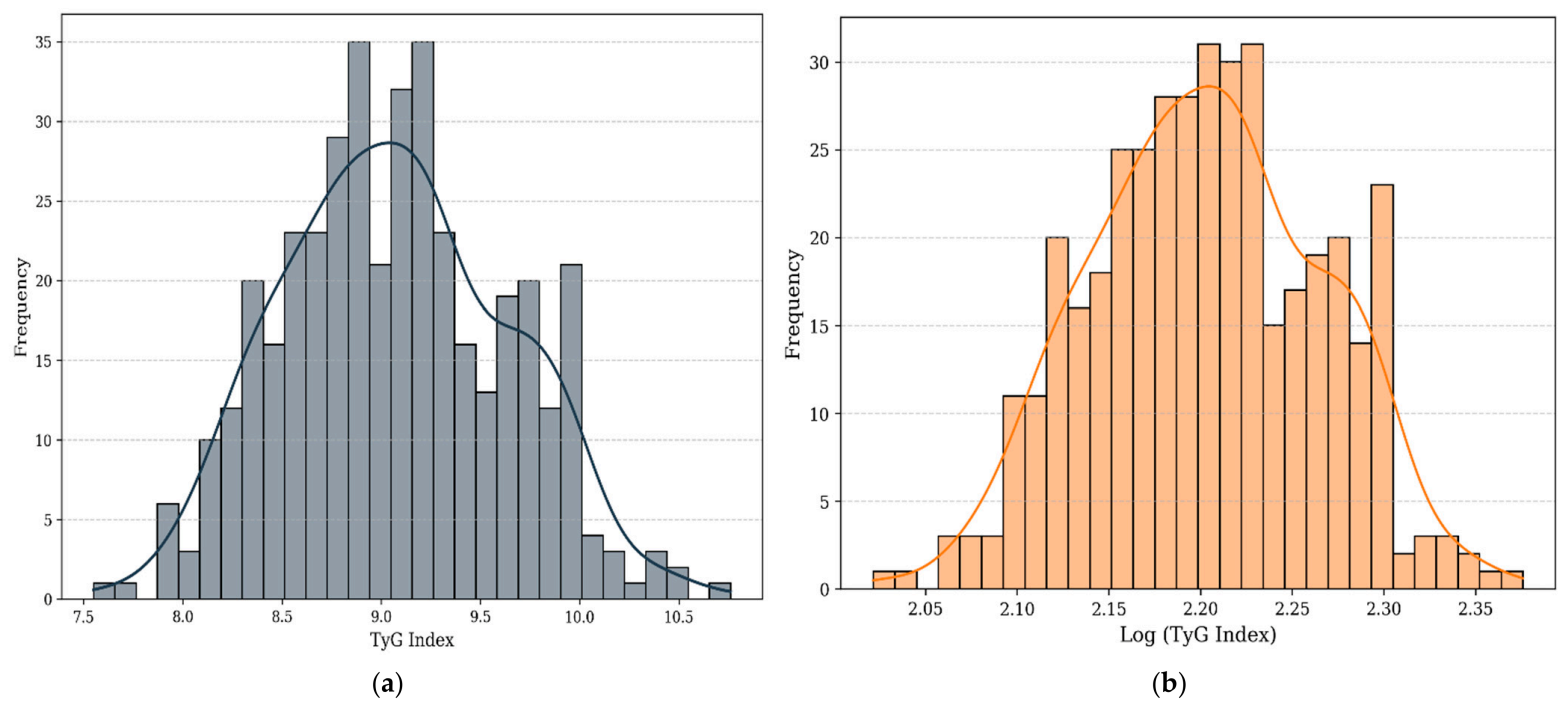
Distribution of TyG index values: (**a**) Raw Data; (**b**) After applying the Log-Transformed framework.

In Figure 4a, the distribution of the TyG index data shows a wide range of variation, with a slight skewness, although not to the same extent as the METS-IR and HOMA-IR scores. A logarithmic transformation was applied to the TyG index values to meet the normality assumption required by parametric tests and to more evenly distribute the variance. In Figure 4b, the resulting TyG index data show a somewhat more consistent distribution.

### 3.4. Feature Selection via Genetic Algorithm

A genetic algorithm (GA) is an optimisation algorithm that models biological evolutionary processes and principles [30,31]. The primary aim of a GA is to explore the solution space by directing models towards regions of the data that show greater potential for optimal performance during feature selection [32]. Introduced by Holland in 1973 [33], GAs—unlike other traditional methods—begin with an initial population consisting of random solutions and represent the solutions found as chromosomes in the form of a sequence of 0 s and 1 s. GAs use natural operators such as mutation, crossover and selection to find the best individuals [34]. Once the initial sets have been determined in a GA, the fitness of each individual is evaluated using a fitness function. Through selection, the fittest individuals in the population become parents, passing on their genes to the next generation. In the crossover operation, the genes of two selected individuals are split at specific points and recombined. The aim here is to produce a child who is significantly better than the two selected individuals. In the mutation operation, changes are made to the genes of randomly selected individuals to maintain diversity. Finally, when the algorithm reaches a specified number of generations, it terminates and outputs the best individual (i.e., the set of features) in the population. In this study, GA was employed to better explore the solution space within a large, high-dimensional dataset. The features influencing the prediction results of the HOMA IR Index, TyG Index and METS IR Index were identified using GA, and these features are presented in Table 3. Upon examining Table 3, it is observed that the glycaemic control parameters Diabetes and Glucose Level were selected by the GA for all three indices. HDL, a strong indicator of metabolic health, was also selected for all three indices. The selection of the Febuxostat parameter across all three scenarios confirms that the dataset comprises patients with gout. Furthermore, these parameters have been shown to influence metabolic indices. In predicting the HOMA-IR index, GA selected inflammatory markers such as AST, ESR, Lymphocyte, and MCV. This suggests that the HOMA-IR index may be closely associated with systemic inflammation. For predicting the TyG index, parameters such as Cerebrovascular Event, Peripheral Artery Disease, Renal Disease, and GFR were selected. This confirms the validity of the TyG index, as the formula used to calculate it includes the Triglyceride parameter and the variables selected by GA reflect cardiovascular and renal risks. For predicting the METS IR index, 25 variables were selected by GA. Notable aspects of the METS IR index, for which the largest number of variables was selected, included data on lifestyle and disease severity. Data such as Cigarette, Alcohol, Tophus, and Flare Frequency were predominantly selected as variables for predicting the METS IR index. The use of BMI to predict the METS IR index and its selection by GA confirms the accuracy of the GA.

### 3.5. Automated Hyperparameter Tuning with Optuna

Optuna is an algorithm that rapidly optimises the hyperparameters at which ensemble and machine learning models exhibit optimal performance, utilising a combination of Bayesian optimisation and other advanced methods to determine these optimal hyperparameter values [35]. It comprises three distinct algorithms: TPE, CMA-ES, and Grid/Random Sampler. In this study, the TPE algorithm was selected because it can optimise categorical and continuous hyperparameters within the dataset’s complexity space using Bayesian inference and because it can rapidly optimise critical hyperparameters of ensemble models, such as tree, leaf, and learning rates [36]. In this way, the hyperparameter space is systematically explored.

The default settings of the models evaluated in this study were not used. To maximize the performance of all models and improve their generalization capabilities, the search spaces shown in Appendix A, along with the optimum hyperparameter values and optimization times found with Optuna for each scenario, are presented in detail in Appendix B (Table A2, Table A3, Table A4, Table A5, Table A6, Table A7, Table A8, Table A9, Table A10, Table A11, Table A12, Table A13, Table A14, Table A15 and Table A16). To achieve high-precision optimisation, a critical search was conducted for Alpha, Lambda, and Gamma—hyperparameters crucial for boosting models—as well as the Learning Rate. For all models, the tree depth (Max Depth) was restricted to 3–9 to balance computational cost and complexity. To ensure that all models achieve their optimal configurations for the dataset, the characteristic features of each boosting model (e.g., Num Leaves for LGBM, loss function for CATBOOST) were incorporated into the optimisation process. The feature selection performed by the GA reflects statistical results based on the variables in the dataset. The feature selection carried out by the GA does not imply biological causality or have definitive clinical significance. However, the GA has identified subsets of features within the dataset that show a strong statistical correlation with the known pathophysiological mechanisms of insulin resistance.

### 3.6. Clinical Prediction Models: Ensemble-Based Regression Approaches

In this study, five ensemble learning models were used to estimate HOMA IR, METS IR, and TyG index values. The AdaBoost, XGB, LGBM, GBM, and CATBOOST models used in this study were selected based on their success in studies of biomedical data and their high validation scores [16,17,18,20]. AdaBoost, a model frequently used in the literature, attempts to make predictions by reweighting weak learners that have made incorrect predictions on randomly generated training subsets [37]. Although the XGB, GBM, and LGBM models are members of the same family, XGB and LGBM were developed from GBM. In the GBM model, trees are constructed sequentially, and each tree corrects the gradient loss of the previous trees [38]. XGB, on the other hand, is a more advanced and powerful version of GBM that constructs trees in parallel. The training process, which begins with an initial prediction, calculates the residuals between the prediction and the observation, and enhances predictive power by minimising these residuals using a second-order Taylor expansion [39]. LGBM is a histogram-based model [40]. LGBM approximates residuals using a second-order Taylor expansion and performs leaf-based growth [41]. Unlike other boosting models, CatBoost processes categorical data and employs a rank-boosting method to handle dataset noise, thereby preventing gradient vanishing and striving to achieve the best predictions by constructing a symmetric model structure [42].

### 3.7. Experimental Design and Scenarios

Within the scope of this study, multi-stage experimental frameworks were implemented to evaluate the performance of ensemble learning models in predicting the HOMA-IR, METS-IR and TyG indices. The aim of creating experimental datasets was to systematically investigate the effects of feature selection using genetic algorithms (GA), in conjunction with statistical data enhancement, on prediction accuracy. Information regarding the experimental design is summarised in Table 4, with the details as follows:

i. Raw Data (Scenario 1): This refers to the scenario in which models are trained and tested on the original dataset, to which no pre-processing or transformations have been applied. In subsequent stages, it serves as a baseline to observe potential improvements in the models.
ii. Log Transformed (Scenario 2): As metabolic clinical data generally do not follow a normal distribution, tend to have high variance, and the target data exhibit right-skewness, a log transformation has been applied to the target data.
iii. GA + Raw Data (Scenario 3): In this scenario, the data dimension was reduced using a genetic algorithm to identify the most relevant clinical data. The raw data, consisting of 55 features, was reduced to between 19 and 25 features depending on the target variable, and the dataset was further reduced to a subset aimed at excluding noisy data from the training set.
iv. GA + Log-Transform (Scenario 4): In this scenario, the genetic algorithm feature selection is combined with a log transformation of the target data.

To ensure the generalisation capability of the ensemble models, the dataset comprising 405 samples was split into an 80% training set and a 20% test set. A random sampling strategy based on target variable quartiles was used to split the training and test datasets. Additionally, to prevent overfitting during the optimization of model hyperparameters using Optuna, 5-fold cross-validation was applied to the training set. Table 5 details the number of features alongside the sample sizes for all experimental scenarios.

### 3.8. Experimental Setup

In this study, training the predictive models, hyperparameter optimisation, and performance evaluation were carried out on Google Colab (Google LLC, Mountain View, CA, USA), a cloud-based platform. The hardware components utilised included an Intel(R) Xeon(R) CPU @ 2.20 GHz (Intel Corporation, Santa Clara, CA, USA), 12.7 GB of LPDDR4 RAM (Micron Technology, Inc., Boise, ID, USA), 6 GB of disk space, and an NVIDIA Tesla T4 (16 GB GDDR6) GPU accelerator (NVIDIA Corporation, Santa Clara, CA, USA). The NumPy (v1.23.5) and Pandas (v1.5.3) libraries were utilised for multi-dimensional matrix operations, whilst the Scikit-Learn (v1.2.2) library was employed for basic regression metrics and cross-validation procedures. PyGAD (v2.18.1) was used for the genetic algorithm, whilst the Matplotlib (v3.7.1) and Seaborn (v0.12.2) libraries were used for visualisation and interpretability; SHAP (SHapley Additive exPlanations) (v0.41.0) was used to interpret the models’ outputs in clinical terms.

### 3.9. Evaluation Metrics

To evaluate the performance of ensemble models, $R^{2}$, $D^{2}$, MSE, MAE, RMSE, MedAE, and MAPE scores were examined; the formulas for these scores are shown in Equations (1), (2), (3), (4), (5), (6) and (7) respectively.

(1) $$R^{2}=1-\frac{\sum_{İ=1}^{N}{\left(y_{i}-{\hat{y}}_{i}\right)}^{2}}{\sum_{İ=1}^{N}{\left(y_{i}-\tilde{y}\right)}^{2}}$$

(2) $$D^{2}=1-\frac{\sum_{i=1}^{N}\left|y_{i}-{\hat{y}}_{i}\right|}{\sum_{i=1}^{N}\left|y_{i}-\bar{y}\right|}$$

(3) $$MSE=\frac{1}{N}\sum_{i=1}^{N}{\left(y_{i}-{\hat{y}}_{i}\right)}^{2}$$

(4) $$MAE=\frac{1}{N}\sum_{i=1}^{N}\left|y_{i}-{\bar{y}}_{i}\right|$$

(5) $$RMSE=\sqrt{\frac{1}{N}\sum_{i=1}^{N}{\left(y_{i}-{\hat{y}}_{i}\right)}^{2}}$$

(6) $$MedAE=median\left(\left|y_{i}-{\hat{y}}_{i}\right|\right)$$

(7) $$MAPE=\frac{100}{N}\sum_{i=1}^{N}\left|\frac{y_{i}-{\hat{y}}_{i}}{y_{i}}\right|$$

In Equations (1)–(7), the symbol N, denotes the numbers of observations; $y_{i}$, denotes the actual value; ${\hat{y}}_{i}$, denotes the predicted value; $\bar{y}$, denotes the mean value and $\tilde{y}$ denotes the median value.

## 4. Results

### 4.1. Model Optimizations and Hyperparameters

The automated hyperparameter tuning process for all five ensemble learning models (XGB, GBM, CatBoost, AdaBoost, and LGBM) across the four experimental scenarios was executed using Optuna’s Tree-structured Parzen Estimator (TPE) algorithm. To maintain structural balance and prevent overfitting, architectural limits such as constraining tree depths (Max Depth) to 3–10 and dynamically adjusting learning rates were applied. Detailed optimal hyperparameter configurations, regularisation parameters (e.g., L2 Leaf Reg, Gamma), and specific base estimator selections determined for each model in predicting HOMA-IR, METS-IR, and TyG indices are provided in Appendix B (Table A2, Table A3, Table A4, Table A5, Table A6, Table A7, Table A8, Table A9, Table A10, Table A11, Table A12, Table A13, Table A14, Table A15 and Table A16). Notably, CatBoost preferred a ‘Symmetric Tree’ growth policy in most scenarios, which enhanced its generalisation capability and resistance to clinical noise. In addition, the search optimisation times and search costs for all models are included in these tables.

### 4.2. Performance Analysis of HOMA-IR Index Prediction

This section of the study presents the results obtained by combining the model architecture with data engineering processes to estimate the HOMA-IR index, as shown in Table 6. Clinical parameters such as the HOMA-IR index naturally exhibit a right-skewed distribution. Consequently, the significant reduction in error metrics observed in the log-transformed scenarios, compared with the raw data set, indicates that the outliers in the data set have been stabilised by the log transformation. This demonstrates the need for statistical normalisation of clinical parameters in clinical datasets. One notable finding in estimating the HOMA-IR index was the effect of the Genetic Algorithm on the models. The GA + Log-Transformed scenario had a positive effect on all models, helping them achieve optimal performance. This demonstrates that right-skewness is corrected by the logarithmic transformation and that the minimum error rate is achieved more effectively with the GA.

Among the ensemble models evaluated for predicting the HOMA-IR index, CATBOOST yielded the lowest errors, with MSE = 0.0008, RMSE = 0.0291, and MAE = 0.0110, alongside the highest predictive accuracy in the GA + Log-Transformed scenario. The CATBOOST model’s symmetric tree architecture and sequential boosting feature enabled it to capture the non-linear structure of the HOMA-IR index without overfitting in the log-transformed scenario, making it the best model. Table 6 also illustrates the sensitivity of the ensemble models to the data structure.

Looking at the ADABOOST model, whilst it exhibited the lowest performance among all models in the Raw Data scenario, it had the lowest RMSE, it improved in the other scenarios. The XGB, GBM, and LGBM models showed improvements depending on the scenario. In particular, the fact that the LGBM and XGB models have similar RMSE and MAPE values in the log-transformed scenarios indicates that these models produce similar results on homogeneously distributed data. In conclusion, the CATBOOST model stands out as the most reliable model for predicting the HOMA-IR index value, a sensitive indicator of insulin resistance. The low error values demonstrated by the model serve as evidence of its potential for use in clinical decision support systems.

### 4.3. Performance Analysis of METS-IR Index Prediction

Table 7 presents the performance results of the models in predicting the METS-IR index across all scenarios. An examination of the models’ performance results shows that, in the log-transformed scenarios, all models exhibit a significant reduction in error metrics compared to the Raw Data scenarios. This statistical analysis demonstrates that the non-linear relationships among the components of the METS-IR formula enable the models to learn more effectively in log-transformed scenarios. Another noteworthy point in Table 7 is the effect of GA-based feature selection on the Raw Data. Although the models generally achieve the lowest error rates in the log-transformed scenarios, it is noteworthy that the CATBOOST model achieves high $R^{2}=0.9890$ and $D^{2}=0.9153$ values in the GA + Raw Data scenario. This indicates that, when predicting the METS-IR index from the original data without any transformation, the model is effective at modelling complex relationships. Another noteworthy point is that in scenarios where a log transformation was applied, the models’ RMSE values decreased significantly, and this decrease was supported by the MAPE values, which are a scale-independent metric. The fact that MAPE values have fallen to the 0.5% level indicates that the multiplicative effect and right-skewness in the METS-IR index formula have been linearised. This serves as proof that a stable space has been created, minimising the models’ prediction errors. The results obtained present two distinct strategies for a clinical decision support system designed to predict the METS-IR index. The first involves training the data using the CATBOOST model by applying the GA + Raw Data scenario, with the aim of accounting for high levels of clinical variance and anomalies in the patient data within the original dataset. The second involves training the data using CATBOOST by applying the GA + Log-Transformed scenario when the objective is to minimise the percentage error margin in predictions.

### 4.4. Performance Analysis of TyG Index Prediction

The findings on the models’ performance in predicting the TyG index are presented in Table 8. Upon examining the analysis results of the models, the most striking point is that all models achieved an $R^{2}$ value above 0.97. The primary reason for obtaining such high results lies in the TyG index formula. The TyG index is a logarithmic function of triglyceride and fasting blood glucose values. Consequently, rather than identifying biological patterns in TyG index prediction, the models have focused on the mathematical relationship between clinical parameters and the target variable and have mapped it with near-perfect accuracy.

The success demonstrated by all models in scenarios where the Log-Transformed transformation was applied provides evidence that the data pre-processing step is compatible with the target variable. The fact that the MSE values in the Log-Transformed and GA + Log-Transformed scenarios have four zeros after the decimal point, whilst the MAPE values have dropped to one-thousandth of a percent, statistically proves that the model does not merely predict, but simulates by copying the formula. In conclusion, the performance of all models constitutes a case study demonstrating the distribution of TyG index data and the power of compatible data transformation. The error metrics of the prediction model using CATBOOST in the scenario where the GA + Log-Transformed transformation was applied demonstrate that this scenario yields reliable results.

## 5. Model Interpretability

The SHAP framework was used to interpret the decision-making mechanisms of the models that yielded the best results in the study. Because the best-performing models were trained on target variables subjected to a natural logarithmic transformation, the SHAP values quantify feature contributions directly on the logarithmic scale. To contextualize these values in terms of raw clinical metrics, feature contributions on the logarithmic scale represent relative, proportional changes in the target variable.

### 5.1. Global and Local Explanations with SHAP for HOMA-IR Index

Figure 4 shows the SHAP plot illustrating the decision-making mechanism of the CATBOOST model, which yielded the best results in the “GA + Log-Transformed” scenario—the scenario that best predicts the HOMA-IR index. The absolute dominance of the Insulin Level and Glucose Level variables in the model’s decision-making process stands out as the most clinically significant finding. Although the formula used to calculate the HOMA-IR index depends on these two variables, the model assigns more than twice the weight to insulin levels compared to glucose levels, reflecting a critical clinical reality. In the early stages of insulin resistance, the body secretes excessive amounts of insulin to maintain normal glucose levels (normoglycaemia). Consequently, the data on insulin levels from patients in the dataset exhibit a wider range of variation compared to the data on glucose levels. In the GA + Log-Transformed scenario, the CATBOOST model’s recognition of biological variance asymmetry and its prioritisation of insulin levels in HOMA-IR estimation demonstrate that the model captures a deep clinical pattern. The model’s explanatory power, which is close to the 2% level, is distributed between the HbA1c and BMI variables. The fact that HbA1c reflects glycaemic control and that BMI is a key physical risk factor for insulin resistance aligns with the model’s logical architecture. The contributions of PLT, HDL, LDL, Neutrophil and vital status (Alive or Dead) are at minimum levels, indicating that the model focuses on pathophysiological variables rather than biomarkers. In conclusion, the SHAP analysis of the CATBOOST model has transformed it into a clinical decision support system by bringing model accuracy to a transparent and reliable level in the GA + Log-Transformed scenario.

**Figure 5:**
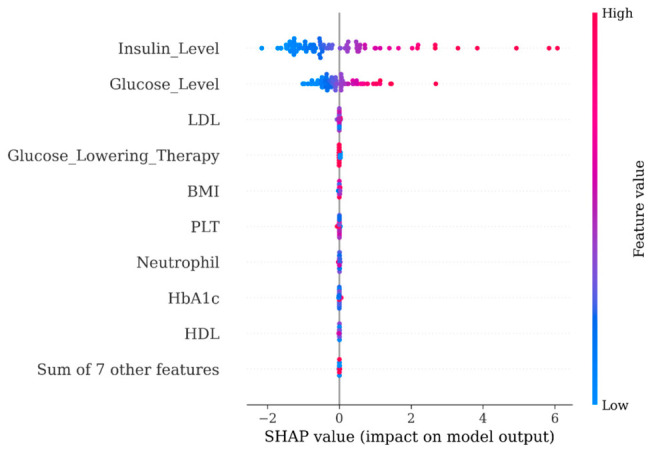
SHAP analysis for CATBOOST in the HOMA-IR index.

The detailed data from the SHAP analysis, illustrated in Figure 5, are presented in Table 9.

### 5.2. Global and Local Explanations with SHAP for METS-IR Index

**Figure 6:**
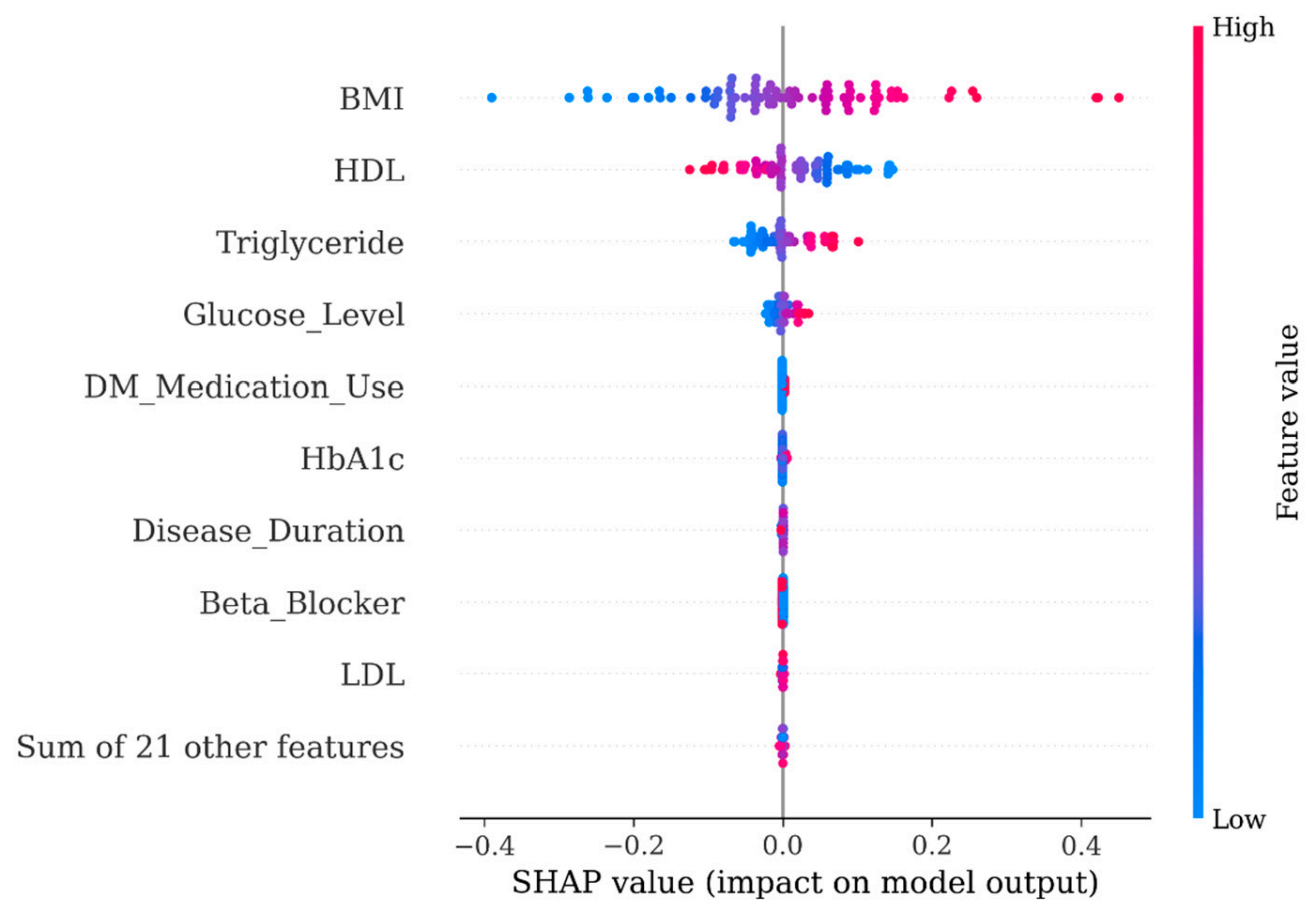
SHAP analysis for CATBOOST on the METS-IR index for the first scenario.

The CATBOOST model, which emerged as the leading model in the GA + Raw Data and GA + Log-Transformed scenarios for METS-IR index prediction, offered two distinct strategies for interpreting the results. The first strategy involves applying the GA + Raw Data scenario when the aim is to account for clinical variance and anomalies in the patient data within the original dataset to a high degree. The results of the SHAP analysis following the application of this strategy are presented in Table 10. Table 10 contains the SHAP importance levels for the GA + Raw Data scenario, which explains the variance and anomalies in the original patient data. Upon examining the analysis results, the most critical finding is that BMI, with a significance level of 52.29%, dominates the model’s prediction process. In this scenario, where no processing was applied to the target data, the patients’ obesity spectrum and other physical anomalies have been fully preserved. When feature selection was performed using GA, the strongest signal also originated from BMI. Upon careful examination of Table 10, it is observed that whilst the HDL and Triglyceride parameters have an effect of approximately 39% after BMI, the effect of Glucose is at the 5.40% level. This indicates that the CATBOOST model makes predictions by relegating glycaemic parameters to the background and shifting the focus of prediction to the BMI–HDL–Triglyceride triangle. The fact that the CATBOOST model derives 96.78% of its predictive power for the METS-IR index from the parameters BMI, HDL, Triglycerides and Glucose Level—which are also included in the METS-IR formula—is noteworthy. It is observed that the model makes predictions without being misled by spurious correlations in noisy data regarding other clinical parameters. The detailed data from the SHAP analysis are illustrated in Figure 6.

**Figure 7:**
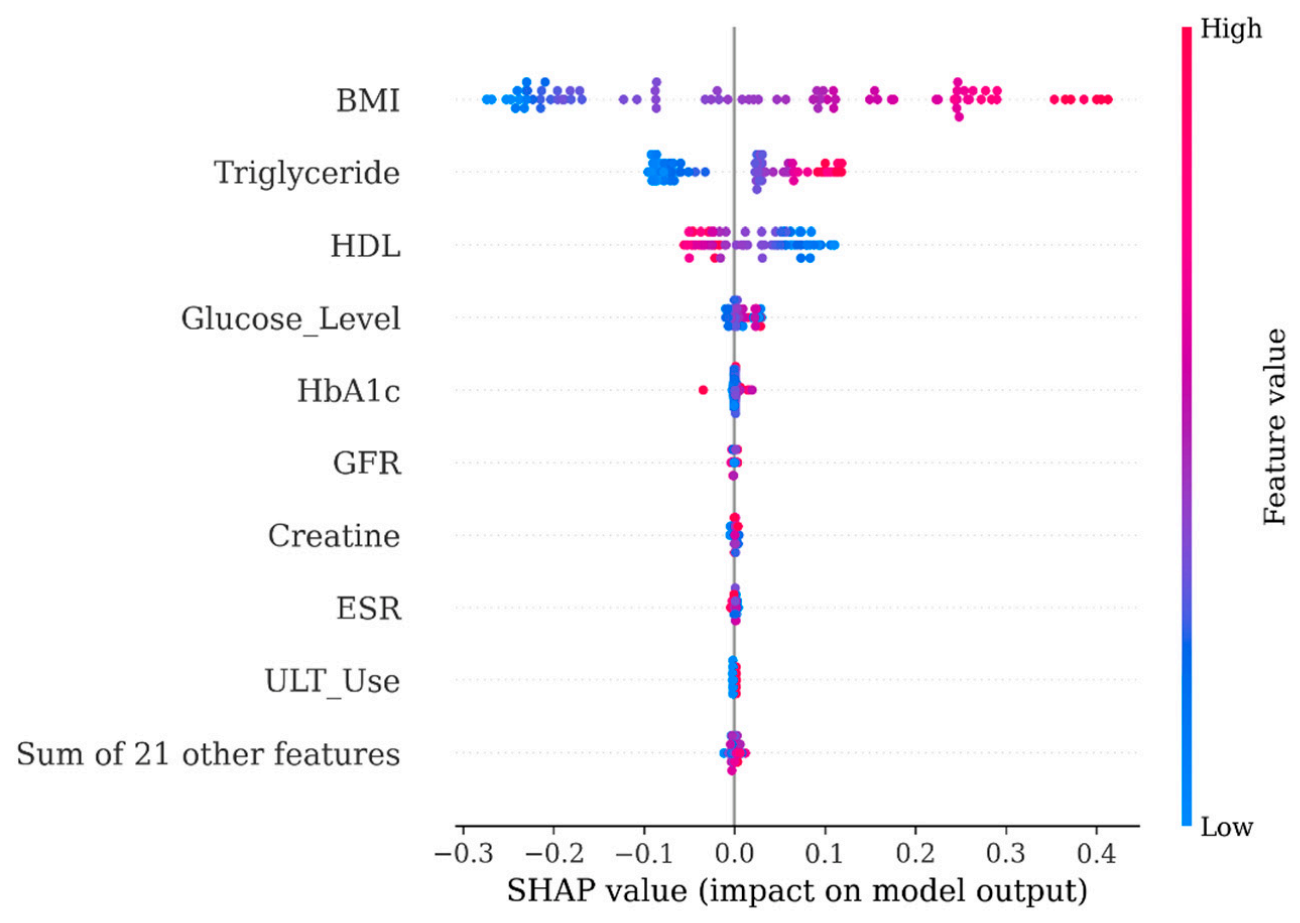
SHAP analysis for CATBOOST on the METS-IR index for the second scenario.

The second strategy involves applying the GA + Log-Transformed scenario when the aim is to minimise the percentage error margin in the predictions. Table 11 shows the SHAP analysis results for the GA + Log-Transformed scenario under this strategy. This strategy demonstrates the statistical impact of the model’s decision mechanism after applying the logarithmic transformation to the target variable. In the GA + Raw Data scenario, Triglyceride, which had a significance level of 14.05%, increased its impact to 20.46% following the logarithmic transformation, thereby becoming the second most important parameter. This aligns with the characteristics of real clinical data for the Triglyceride parameter. Whilst triglycerides exhibit a right-skewed distribution in patient data, HDL shows a narrower range of variation. The application of a log-transformation demonstrates that the linear relationship between Triglyceride and METS-IR is captured smoothly. Consequently, the prediction error (MAPE) has been reduced to very low levels. Following the application of a log-transformation to the target variable, BMI retained its first-order importance whilst increasing its significance level to 57.18%. With the increase in the significance of BMI, CATBOOST has mapped the pathophysiological mechanism more accurately. Whilst GFR, Creatinine, and ESR parameters remained in the background in the first strategy (GA + Raw Data scenario), they are observed to influence the model’s decision-making mechanism in the second strategy (GA + Log-Transformed scenario). It is well established in the medical literature that impairment of renal function due to insulin resistance is strongly correlated with inflammation. In the second strategy, the model has minimised prediction errors by considering patients’ renal and inflammatory changes. The detailed data from the SHAP analysis are illustrated in Figure 7.

### 5.3. Global and Local Explanations with SHAP for TyG Index

**Figure 8:**
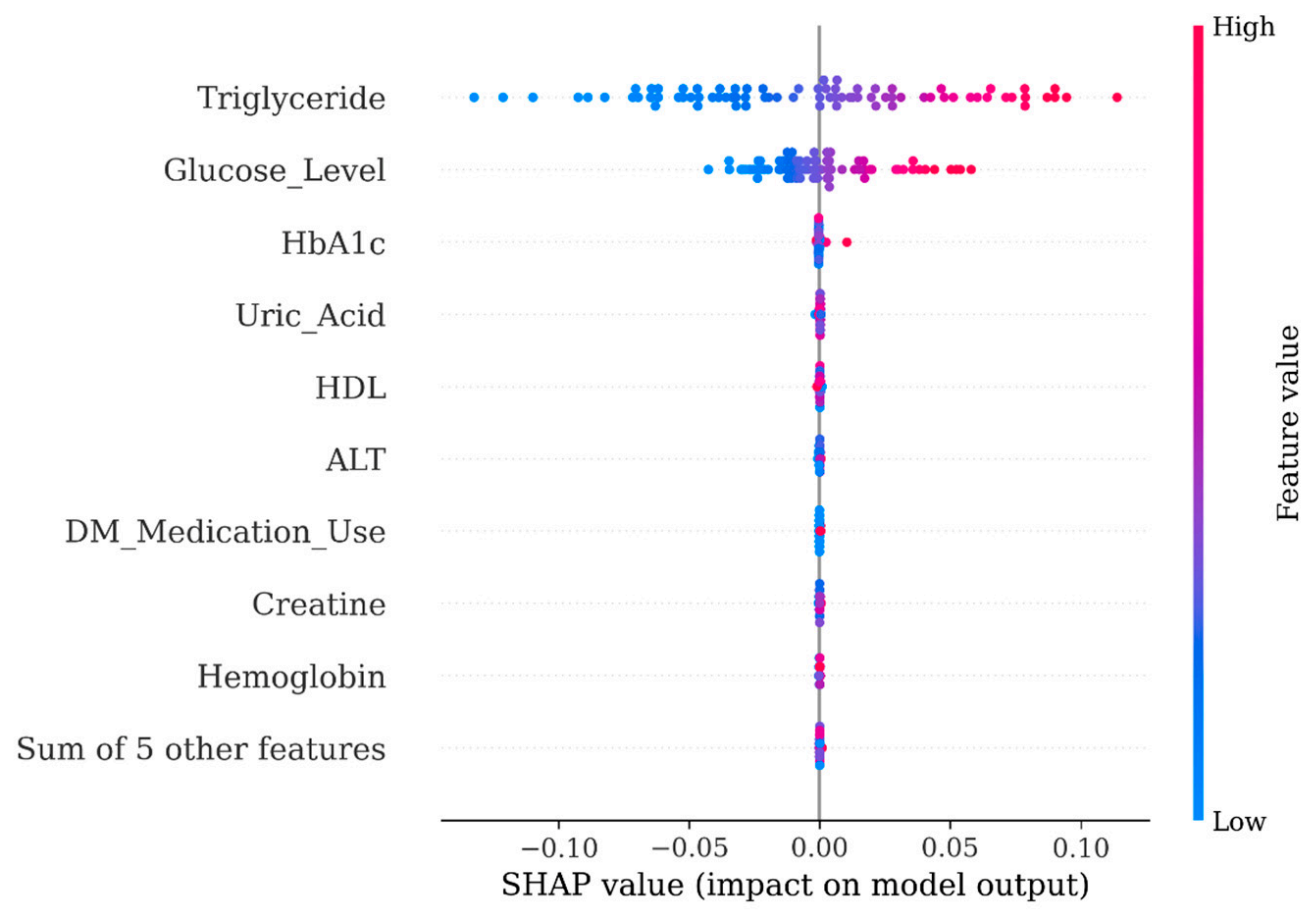
SHAP analysis for CATBOOST in TyG index.

Table 12 presents the SHAP analysis results for the CATBOOST model in the GA + Log-Transformed scenario, one of four scenarios used for TyG index prediction. The most striking finding is that the Triglyceride and Glucose Level parameters dominate the model’s decision-making process. Together, their influence accounts for 97.45%. Because these two parameters are used in the TyG index formula and retain high influence even after GA feature selection, this indicates that the model does not overfit; on the contrary, it captures mathematical and biological reality. Although Triglyceride and Glucose levels are the two main factors in the TyG formula, the Triglyceride parameter’s 70.60% importance level provides critical statistical and clinical validity. In human metabolism, Glucose is regulated by homeostatic mechanisms, resulting in a narrow fluctuation range for Glucose levels. Triglyceride, on the other hand, fluctuates over a wide range due to dyslipidaemia, in stark contrast to Glucose. The CATBOOST model’s ability to detect this clinical asymmetry in the data, and its mathematical and statistical identification of triglycerides as the parameter driving the primary change in the TyG index value, confirms the model’s superior performance. Additionally, its ability to capture subtle fluctuations and deviations in glucose levels through HbA1c represents a notable advancement. The detailed data from the SHAP analysis are illustrated in Figure 8.

This study demonstrates the adaptive intelligence of the CATBOOST model when applied in the GA + Log-Transformed scenario. The CATBOOST model has effectively reverse-engineered the pathophysiological anatomy of three distinct medical formulas in the dataset—focusing on Insulin for HOMA-IR index prediction, BMI for METS-IR prediction, and Triglyceride and Glucose Level for TyG Index prediction—without deviating from clinical focal points.

## 6. Discussion

This study aimed to predict HOMA-IR, METS-IR, and the TyG Index—three key indices used in the clinical diagnosis of insulin resistance—using ensemble learning models. The findings from training and optimising the models highlight the role of logarithmic transformation—which aligns with the nature of biological parameters—and feature selection based on Genetic Algorithms in the models’ predictive performance. Unlike previous studies that focus on predicting a single index, this study presents a methodological approach that tests three distinct indices with different mathematical structures within the same pipeline. One of the study’s most critical findings is that the Log-Transformed scenario significantly reduces error metrics across all models, bringing them close to zero.

The study’s most original contribution to the literature is its use of SHAP analysis to elucidate how the decision mechanism of the CATBOOST model—which emerged as the most successful—operates. In predicting the HOMA-IR index, the CATBOOST model identified variance in rising insulin levels as the key driver of resistance in the early stages, thereby helping ensure that the body’s glucose levels remain within healthy reference ranges. In predicting the METS-IR index, the model shifted decision weights towards obesity-related variables (BMI, Triglycerides, HDL), setting glycaemic parameters aside, thereby validating the index’s characterisation of dyslipidaemia. Finally, in predicting the TyG Index, the model identified triglycerides—which exhibit a wide distribution—as the primary source of variance, rather than glucose, which shows a narrow distribution, and successfully simulated the mathematical formula used in TyG Index calculations with near-perfect accuracy. However, rather than establishing biological causality—which Ensemble models cannot directly prove—all these results —by emphasizing the prioritization of features such as insulin via GA and SHAP analyses, BMI for METS-IR, and triglycerides for the TyG index—demonstrate that the model yielding the best results effectively utilizes prediction patterns that closely reflect pathophysiological mechanisms. The findings reveal that the model captures statistical synergies based on clinical data. In routine clinical practice, threshold values for metabolic indices are used to identify insulin resistance: HOMA-IR ≥ 2.5, METS-IR ≥ 41.33, and TgY index ≥ 8.85. Based on these threshold values, diagnostic thresholds can be integrated between regression outputs and clinical decision-making. For example, automatic alerts such as “High Metabolic Risk” or “Insulin Resistance Present” could be provided within Hospital Information Systems.

In recent years, there has been a growing body of research on predicting insulin resistance using artificial intelligence methods. In our study, the CatBoost model’s superior performance across all indices supports the notion that gradient-boosting algorithms can effectively model the complex, nonlinear biological relationships associated with insulin resistance. However, unlike the current study, the focus was not limited to a single index; the HOMA-IR, METS-IR, and TyG indices were evaluated simultaneously within the same data-processing pipeline, and the contributions of logarithmic transformation and genetic algorithm-based feature selection were examined in detail.

The literature shows that the HOMA-IR and TyG indices are key predictors of metabolic diseases, and that biologically meaningful variables emerge when using artificial intelligence methods. In our study, the SHAP results show a high degree of consistency with the mathematical structures of these indices. The fact that the CATBOOST model selected insulin as the dominant predictor for HOMA-IR, BMI and triglycerides for METS-IR, and triglycerides for TyG indicates that the model not only learns statistical patterns but also successfully reflects the underlying biological mechanisms of insulin resistance. Comparative results between the findings obtained in this study and those reported in the literature are presented in Table 13.

### Strengths and Limitations

This study has several significant strengths. First, the HOMA-IR, METS-IR, and TyG indices—which are commonly used to assess insulin resistance—were evaluated simultaneously within the same data-processing and modelling pipeline, thereby enabling a direct comparison of their performance despite their differing mathematical and biological structures. Second, by combining logarithmic transformation, Genetic Algorithm-based feature selection, ensemble learning algorithms, and SHAP-based explainable AI methods, both high predictive performance and clinical interpretability were achieved. In particular, the variable importance rankings revealed by SHAP analyses align with the pathophysiological foundations of the indices, demonstrating that the models capture not only statistical relationships but also clinically meaningful patterns. However, the study also has some limitations. The study was conducted using a retrospective design, and data were obtained from a single centre. Due to the retrospective data collection design, patients’ daily dietary habits, purine-rich food intake, or Mediterranean diet adherence scores are not recorded in medical archive records using a standardized scale. This may limit the generalisability of the results to different ethnic groups and populations. Before use in clinical settings, external validation across diverse ethnic groups and populations is essential. In addition, the HOMA-IR, METS-IR and TyG indices, which were used as outcome variables in this study, are indirect markers of insulin resistance. It was not possible to compare these with the hyperinsulinaemic-euglycaemic clamp technique, which is regarded as the gold standard in rheumatology settings due to its invasive nature and the routine outpatient setting. Consequently, these findings need to be supported by future multicentre, prospective studies involving external validation.

## 7. Conclusions

Considering the entire study, four dataset scenarios were created to estimate HOMA-IR, METS-IR, and TyG index values, and comparative analyses of the models were conducted for each scenario using different data preprocessing and feature selection methods. Among the ensemble learning models, CATBOOST emerged as the best, achieving the highest R^2^ and D^2^ scores and the lowest error rates when feature selection was performed using Genetic Algorithms and the target variable was log-transformed. It achieved 99.70% accuracy in predicting the TyG index, with explainability nearly perfect. Through SHAP-based Explainable Artificial Intelligence (XAI) analysis, the CATBOOST model was clinically validated as making decisions weighted towards Insulin for HOMA-IR, BMI and Triglycerides for METS-IR, and Triglycerides for TyG, confirming that artificial intelligence is fully aligned with medical causality. Rather than establishing biological causality—which statistical models cannot directly prove—the high priority assigned by GA and SHAP to features such as insulin, BMI and triglycerides demonstrates that CatBoost produces a prediction that is highly consistent with the established pathophysiological mechanisms of insulin resistance. As a result, the CATBOOST pipeline applied in the proposed GA + Log-Transformed scenario has been shown to be a reliable decision support system for the early detection of insulin resistance using the evaluated indices. Future studies aim to test different artificial intelligence models and hybrid models across various demographic characteristics and to measure the prospective clinical benefit by integrating systems with real-time data streams into hospital information systems.

## Figures and Tables

**Table 1:** Demographic and clinical characteristics of the study population.

Variables		Male	Female	Total
Demographics	Number of Patients	283	128	412
	Age (Years), mean ± SD	64.48 ± 12.49	72.41 ± 10.50	66.99 ± 12.45
	Sex	283 (68.70%)	128 (31.10%)	412 (100%)
	BMI $\left(kg/m^{2}\right)$, mean ± SD	31.27 ± 16.56	33.27 ± 5.77	31.87 ± 14.13
Lifestyle	Smoking, n (%)	43 (15.20%)	1 (0.8%)	44 (10.70%)
	Alcohol Consumption, n (%)	30 (10.6%)	2 (1.6%)	32 (7.8%)
Disease Baseline	Disease Duration (years), median(IQR)	65.31 ± 46.88	59.74 ± 28.94	63.51 ± 42.14
	Family History of Gouty, n (%)	98 (34.6%)	36 (28.1%)	134 (32.5%)
Comorbidities	Hypertension, n (%)	236 (83.4%)	122 (95.3%)	359 (87.1%)
	Diabetes Mellitus, n (%)	72 (25.4%)	58 (45.3%)	130 (31.6%)
	Chronic Kidney Disease, n (%)	78 (27.6%)	62 (48.4%)	141 (34.2%)

**Table 2:** Detailed clinical parameters, laboratory values, and medication regimens of the study group.

Variable	Male (Median [IQR])	Female Male (Median [IQR])	Total (Median [IQR])	*p*-Value (MWU)
ACE/ARB Use	0.00 [0.00–1.00]	0.00 [0.00–0.00]	0.00 [0.00–0.00]	0.0211
Age	66.00 [56.00–74.00]	74.00 [66.00–80.00]	68.00 [59.75–76.00]	<0.0001
Alcohol	0.00 [0.00–0.00]	0.00 [0.00–0.00]	0.00 [0.00–0.00]	0.0016
Alive or Dead	1.00 [1.00–1.00]	1.00 [1.00–1.00]	1.00 [1.00–1.00]	0.0994
ALT	24.00 [17.00–36.00]	18.00 [13.00–30.00]	22.50 [15.00–35.00]	<0.0001
Antidiabetic Drug Use	0.00 [0.00–0.00]	0.00 [0.00–1.00]	0.00 [0.00–1.00]	<0.0001
AST	23.00 [19.00–30.00]	20.50 [17.00–28.00]	22.00 [18.00–30.00]	0.0343
Attack Quiescent Period	0.00 [0.00–1.00]	0.00 [0.00–1.00]	0.00 [0.00–1.00]	0.0224
Beta Blocker	0.00 [0.00–1.00]	0.00 [0.00–1.00]	0.00 [0.00–1.00]	0.4819
BMI	29.63 [27.77–32.53]	32.10 [29.64–35.52]	30.38 [28.09–33.54]	<0.0001
BPH	0.00 [0.00–0.00]	0.00 [0.00–0.00]	0.00 [0.00–0.00]	0.0001
CAD	0.00 [0.00–1.00]	0.00 [0.00–0.00]	0.00 [0.00–1.00]	0.0258
CCBs	0.00 [0.00–1.00]	0.00 [0.00–1.00]	0.00 [0.00–1.00]	0.0209
CE	0.00 [0.00–0.00]	0.00 [0.00–0.00]	0.00 [0.00–0.00]	0.7802
CHF	0.00 [0.00–0.00]	0.00 [0.00–0.00]	0.00 [0.00–0.00]	0.2517
Cholesterol	202.00 [176.50–243.00]	219.00 [179.75–244.25]	209.00 [178.00–244.00]	0.0824
Cigarette	0.00 [0.00–0.00]	0.00 [0.00–0.00]	0.00 [0.00–0.00]	<0.0001
Colchicine	0.00 [0.00–1.00]	1.00 [0.00–1.00]	0.00 [0.00–1.00]	0.0257
Corticosteroids	0.00 [0.00–0.00]	0.00 [0.00–0.00]	0.00 [0.00–0.00]	0.3731
Creatine	1.09 [0.90–1.29]	1.00 [0.80–1.20]	1.07 [0.90–1.27]	0.0023
CRP	5.70 [2.40–10.50]	7.00 [3.75–16.25]	6.00 [2.70–12.93]	0.0083
Diabetes	0.00 [0.00–1.00]	0.00 [0.00–1.00]	0.00 [0.00–1.00]	0.0001
Disease Duration	60.00 [36.00–72.00]	60.00 [48.00–72.00]	60.00 [36.00–72.00]	0.6528
Diuretic Use	0.00 [0.00–1.00]	0.00 [0.00–1.00]	0.00 [0.00–1.00]	0.0007
Dyslipidaemia	0.00 [0.00–1.00]	0.00 [0.00–1.00]	0.00 [0.00–1.00]	0.1368
ESR	13.00 [7.00–22.00]	22.00 [13.00–38.25]	16.00 [9.00–28.00]	<0.0001
Family Have Gouty	0.00 [0.00–1.00]	0.00 [0.00–1.00]	0.00 [0.00–1.00]	0.1934
Febuxostat	0.00 [0.00–0.00]	0.00 [0.00–0.00]	0.00 [0.00–0.00]	0.5606
First Attack Age	59.00 [50.00–67.50]	67.50 [59.00–74.00]	62.00 [53.00–70.00]	<0.0001
Flare Frequency	2.00 [2.00–3.00]	2.00 [2.00–3.00]	2.00 [2.00–3.00]	0.7933
GFR	74.00 [58.00–90.00]	59.00 [45.00–78.25]	70.00 [54.00–87.00]	<0.0001
Glucose Level	102.00 [93.00–112.50]	109.00 [91.00–126.25]	103.00 [92.00–117.00]	0.0433
Glucose Lowering Therapy	1.00 [1.00–1.00]	1.00 [0.00–1.00]	1.00 [0.00–1.00]	<0.0001
Haemoglobin	14.00 [13.00–15.00]	12.00 [11.00–13.00]	14.00 [12.00–15.00]	<0.0001
HbA1c	5.90 [5.60–6.10]	6.00 [5.70–6.62]	5.90 [5.60–6.20]	0.0010
HDL	42.00 [36.00–47.50]	48.00 [40.00–54.25]	44.00 [37.00–50.25]	<0.0001
Hypertension	1.00 [1.00–1.00]	1.00 [1.00–1.00]	1.00 [1.00–1.00]	0.0009
Insulin Level	9.00 [6.67–13.00]	9.45 [6.50–13.00]	9.00 [6.60–13.00]	0.6888
LDL	130.00 [101.00–160.75]	129.00 [104.50–158.25]	130.00 [101.00–160.00]	0.6718
Lipid Lowering Therapy	1.00 [0.00–1.00]	1.00 [0.00–1.00]	1.00 [0.00–1.00]	0.2800
Lymphocyte	2310.00 [1755.00–2805.00]	2325.00 [1840.00–2930.00]	2315.00 [1797.50–2840.00]	0.6718
MCV	88.00 [85.00–91.00]	89.00 [86.00–92.00]	88.50 [85.00–92.00]	0.0909
Nephrolithiasis	0.00 [0.00–0.00]	0.00 [0.00–0.00]	0.00 [0.00–0.00]	0.0098
Neutrophil	4600.00 [3,680.00–5855.00]	4515.00 [3430.00–5650.00]	4575.00 [3657.50–5822.50]	0.5061
NSAID	0.00 [0.00–0.00]	0.00 [0.00–0.00]	0.00 [0.00–0.00]	0.4258
PAD	0.00 [0.00–0.00]	0.00 [0.00–0.00]	0.00 [0.00–0.00]	0.8839
PLT	233,000.00 [195,000.00–274,500.00]	249,000.00 [211,000.00–308,750.00]	237,000.00 [197,000.00–281,250.00]	0.0048
Renal Disease	0.00 [0.00–1.00]	0.00 [0.00–1.00]	0.00 [0.00–1.00]	<0.0001
Tophus Count	0.00 [0.00–0.00]	0.00 [0.00–0.00]	0.00 [0.00–0.00]	0.2512
Triglyceride	165.00 [111.50–239.50]	158.00 [113.25–227.50]	162.50 [111.75–232.50]	0.5582
ULT Use	0.00 [0.00–1.00]	1.00 [0.00–1.00]	1.00 [0.00–1.00]	0.0481
Urea	39.00 [31.50–49.00]	40.50 [35.00–56.00]	40.00 [32.00–51.00]	0.0451
Uric Acid	8.20 [7.43–9.20]	8.20 [7.00–9.30]	8.20 [7.30–9.28]	0.3505
WBC	7940.00 [6705.00–9445.00]	7790.00 [6635.00–8967.50]	7915.00 [6680.00–9322.50]	0.4142

To ensure that variables showing no statistically significant differences did not lead to model instability, multicollinearity was assessed prior to modelling using the variance inflation factor (VIF). A threshold of 10 was set for the VIF, and the variables had VIFs < 10, indicating the absence of multicollinearity. Furthermore, the Genetic Algorithm (GA) was used to remove features that did not contribute to the model.

**Table 3:** Features selected using GA for the prediction of the HOMA IR Index, TyG Index and METS IR Index (✓ indicates that the variable was selected by the genetic algorithm (GA), whereas × indicates that the variable was not selected).

Variable	HOMA-IR Index	TyG Index	METS-IR
ACE/ARB Use	×	×	×
Age	×	×	×
Alcohol	×	**✓**	×
Alive or Dead	**✓**	**✓**	×
ALT	×	×	**✓**
Antidiabetic Drug Use	×	**✓**	**✓**
AST	×	×	×
Attack Quiescent Period	**✓**	×	×
Beta Blocker	×	**✓**	×
BMI	**✓**	**✓**	×
BPH	**✓**	×	×
CAD	×	**✓**	×
CCBs	**✓**	**✓**	**✓**
CE	×	×	**✓**
CHF	×	×	×
Cholesterol	×	×	×
Cigarette	**✓**	**✓**	×
Colchicine	×	**✓**	×
Corticosteroids	×	**✓**	×
Creatine	×	**✓**	**✓**
CRP	×	×	×
Diabetes	×	×	**✓**
Disease Duration	×	**✓**	×
Diuretic Use	×	×	**✓**
Dyslipidaemia	×	×	×
ESR	×	**✓**	×
Family Have Gouty	×	**✓**	×
Febuxostat	×	**✓**	×
First Attack Age	×	×	×
Flare Frequency	×	×	**✓**
GFR	×	**✓**	×
Glucose Level	**✓**	**✓**	**✓**
Glucose Lowering Therapy	**✓**	**✓**	×
Haemoglobin	×	**✓**	**✓**
HbA1c	**✓**	**✓**	**✓**
HDL	**✓**	**✓**	**✓**
Hypertension	×	**✓**	×
Insulin Level	**✓**	**✓**	×
LDL	**✓**	**✓**	×
Lipid Lowering Therapy	×	×	×
Lymphocyte	×	×	×
MCV	×	×	×
Nephrolithiasis	**✓**	×	×
Neutrophil	**✓**	×	×
NSAID	×	**✓**	×
PAD	×	**✓**	×
PLT	**✓**	×	×
Renal Disease	**✓**	**✓**	×
Tophus Count	**✓**	**✓**	×
Triglyceride	×	**✓**	**✓**
ULT	×	**✓**	×
Urea	×	×	×
Uric Acid	×	×	**✓**
WBC	×	×	×
Number of Selected Features via GA	16	30	14

**Table 4:** Data set scenarios designed for predicting metabolic insurance scores.

Scenario	Definition	Rationale
Raw Data	No operations were performed on the dataset.	To establish a baseline for comparative analysis.
Log-Transformed	Natural log transformation applied to target variables.	To correct right-skewed distributions and reduce outlier sensitivity.
GA + Raw Data	Feature selection via Genetic Algorithm on the original data	To identify the most influential predictors and reduce dimensionality.
GA + Log-Transformed	Combined GA-based selection and log transformation for target variables.	To optimise both feature relevance and statistical distribution.

**Table 5:** Dataset scenarios designed for estimating metabolic indices.

Prediction	Scenario	Number of Features	Training Sample	Test Sample	Total
HOMA-IR	Raw Data	55	324	81	405
	Log-Transformed	55	324	81	405
	GA + Raw Data	55	324	81	405
	GA + Log-Transformed	55	324	81	405
METS-IR	Raw Data	55	324	81	405
	Log-Transformed	55	324	81	405
	GA + Raw Data	55	324	81	405
	GA + Log-Transformed	55	324	81	405
TyG Index	Raw Data	55	324	81	405
	Log-Transformed	55	324	81	405
	GA + Raw Data	55	324	81	405
	GA + Log-Transformed	55	324	81	405

**Table 6:** A comparative analysis of models in the HOMA-IR index predicting based on data set scenarios.

Model	Scenarios	$R^{2}$	$D^{2}$	MSE	MAE	RMSE	MedAE	MAPE
XGB	Raw Data	0.9815	0.8558	0.0584	0.1647	0.2417	0.1221	0.0615
	Log-Transformed	0.9955	0.9392	0.0012	0.0241	0.0343	0.0179	0.0618
	GA + Raw Data	0.9801	0.8674	0.0627	0.1515	0.2504	0.0915	0.0555
	GA + Log-Transformed	0.9895	0.9214	0.0028	0.0311	0.0527	0.0158	0.1070
GBM	Raw Data	0.9809	0.8912	0.0603	0.1243	0.2455	0.0614	0.0424
	Log-Transformed	0.9957	0.9394	0.0011	0.0240	0.0336	0.0204	0.0557
	GA + Raw Data	0.9784	0.9003	0.0680	0.1139	0.2608	0.0316	0.0375
	GA + Log-Transformed	0.9942	0.9427	0.0015	0.0227	0.0390	0.0115	0.0886
CATBOOST	Raw Data	0.9942	0.9268	0.0183	0.0836	0.1352	0.0485	0.0331
	Log-Transformed	0.9948	0.9396	0.0014	0.0239	0.0372	0.0151	0.0990
	GA + Raw Data	0.9955	0.9310	0.0143	0.0788	0.1197	0.0497	0.0312
	***GA + Log-Transformed***	***0.9968***	***0.9540***	***0.0008***	***0.0182***	***0.0291***	***0.0110***	***0.0527***
ADABOOST	Raw Data	0.8612	0.5778	0.4378	0.4822	0.6617	0.3769	0.2241
	Log-Transformed	0.9694	0.8616	0.0081	0.0549	0.0303	0.0303	0.1623
	GA + Raw Data	0.9583	0.8482	0.1315	0.1733	0.3626	0.0580	0.0586
	GA + Log-Transformed	0.9778	0.8887	0.0059	0.0441	0.0240	0.0240	0.1308
LGBM	Raw Data	0.9552	0.8459	0.1414	0.1761	0.3760	0.0707	0.0575
	Log-Transformed	0.9920	0.9202	0.0021	0.0316	0.0461	0.0200	0.0598
	GA + Raw Data	0.9681	0.8302	0.1005	0.1939	0.3170	0.1069	0.0669
	GA + Log-Transformed	0.9921	0.9308	0.0021	0.0274	0.0456	0.0161	0.0522
MLR	Raw Data	0.9419	0.7643	0.1833	0.2692	0.4282	0.1675	0.1129
	Log-Transformed	0.8971	0.6828	0.0272	0.1258	0.1649	0.0915	0.4089
	GA + Raw Data	0.9589	0.8201	0.1297	0.2054	0.3601	0.1174	0.0826
	GA + Log-Transformed	0.9010	0.6861	0.0262	0.1244	0.1617	0.0950	0.4066

**Table 7:** A comparative analysis of models in the METS-IR index predicting based on data set scenarios.

Model	Scenarios	$R^{2}$	$D^{2}$	MSE	MAE	RMSE	MedAE	MAPE
XGB	Raw Data	0.9700	0.8436	2.2827	1.0334	1.5109	0.7119	0.0192
	Log-Transformed	0.9703	0.8308	0.0008	0.0222	0.0290	0.0180	0.0056
	GA + Raw Data	0.9791	0.8717	1.5870	0.8479	1.2598	0.5032	0.0161
	GA + Log-Transformed	0.9810	0.8704	0.0005	0.0170	0.0232	0.0116	0.0043
GBM	Raw Data	0.9781	0.8702	1.6665	0.8577	1.2909	0.6068	0.0159
	Log-Transformed	0.9793	0.8491	0.0006	0.0198	0.0242	0.0184	0.0050
	GA + Raw Data	0.9871	0.9018	0.9777	0.6491	0.9888	0.4056	0.0121
	GA + Log-Transformed	0.9826	0.8783	0.0005	0.0159	0.0222	0.0112	0.0040
CATBOOST	Raw Data	0.9758	0.8557	1.8408	0.9536	1.3568	0.6195	0.0182
	Log-Transformed	0.9838	0.8804	0.0005	0.0157	0.0214	0.0121	0.0040
	***GA + Raw Data***	***0.9890***	***0.9153***	***0.8331***	***0.5598***	***0.9127***	***0.3400***	***0.0106***
	***GA + Log-Transformed***	***0.9856***	***0.8958***	***0.0004***	***0.0136***	***0.0202***	***0.0090***	***0.0034***
ADABOOST	Raw Data	0.8837	0.6737	8.8384	2.1565	2.9729	1.6600	0.0413
	Log-Transformed	0.9194	0.7220	0.0023	0.0364	0.0478	0.0311	0.0093
	GA + Raw Data	0.9266	0.7383	5.5797	1.7294	2.3621	1.2467	0.0339
	GA + Log-Transformed	0.9388	0.7658	0.0017	0.0307	0.0416	0.0220	0.0078
LGBM	Raw Data	0.9404	0.8142	4.5325	1.2278	2.1290	0.7590	0.0224
	Log-Transformed	0.9519	0.8219	0.0014	0.0233	0.0369	0.0151	0.0058
	GA + Raw Data	0.9600	0.8335	3.0394	1.1002	1.7434	0.6624	0.0203
	GA + Log-Transformed	0.9583	0.8198	0.0012	0.0236	0.0343	0.0159	0.0059
MLR	Raw Data	−0.0544	0.1142	80.1582	5.8535	8.9531	4.1637	0.1169
	Log-Transformed	−0.0126	0.1267	0.0287	0.1144	0.1694	0.0871	0.0292
	GA + Raw Data	0.2522	0.1978	56.8520	5.3010	7.5400	4.1538	0.1042
	GA + Log-Transformed	0.2790	0.1923	0.0204	0.1058	0.1429	0.0850	0.0269

**Table 8:** A comparative analysis of models in the TyG index predicting based on data set scenarios.

Model	Scenarios	$R^{2}$	$D^{2}$	MSE	MAE	RMSE	MedAE	MAPE
XGB	Raw Data	0.9946	0.9394	0.0014	0.0258	0.0381	0.0173	0.0028
	Log-Transformed	0.9951	0.9415	0.0000	0.0028	0.0040	0.0020	0.0012
	GA + Raw Data	0.9956	0.9428	0.0012	0.0243	0.0343	0.0172	0.0027
	GA + Log-Transformed	0.9945	0.9304	0.0000	0.0033	0.0043	0.0026	0.0015
GBM	Raw Data	0.9933	0.9294	0.0018	0.0300	0.0424	0.0222	0.0033
	Log-Transformed	0.9937	0.9306	0.0000	0.0033	0.0046	0.0025	0.0015
	GA + Raw Data	0.9962	0.9404	0.0010	0.0253	0.0321	0.0210	0.0028
	GA + Log-Transformed	0.9956	0.9446	0.0000	0.0026	0.0038	0.0019	0.0012
CATBOOST	Raw Data	0.9953	0.9415	0.0013	0.0248	0.0357	0.0187	0.0028
	Log-Transformed	0.9977	0.9574	0.0000	0.0020	0.0028	0.0014	0.0009
	GA + Raw Data	0.9980	0.9588	0.0005	0.0175	0.0229	0.0143	0.0019
	***GA + Log-Transformed***	***0.9979***	***0.9600***	***0.0000***	***0.0019***	***0.0026***	***0.0014***	***0.0009***
ADABOOST	Raw Data	0.9726	0.8756	0.0073	0.0529	0.0856	0.0329	0.0059
	Log-Transformed	0.9738	0.8621	0.0001	0.0065	0.0093	0.0041	0.0030
	GA + Raw Data	0.9765	0.8680	0.0063	0.0561	0.0794	0.0400	0.0062
	GA + Log-Transformed	0.9776	0.8709	0.0001	0.0061	0.0086	0.0039	0.0028
LGBM	Raw Data	0.9899	0.9187	0.0027	0.0346	0.0519	0.0224	0.0038
	Log-Transformed	0.9927	0.9195	0.0000	0.0038	0.0049	0.0031	0.0017
	GA + Raw Data	0.9956	0.9370	0.0012	0.0268	0.0341	0.0224	0.0029
	GA + Log-Transformed	0.9954	0.9362	0.0000	0.0030	0.0039	0.0023	0.0014
MLR	Raw Data	0.9201	0.7380	0.0214	0.1113	0.1462	0.0945	0.0125
	Log-Transformed	0.9045	0.7132	0.0003	0.0135	0.0178	0.0110	0.0062
	GA + Raw Data	0.9271	0.7473	0.0195	0.1074	0.1397	0.0908	0.0120
	GA + Log-Transformed	0.9113	0.7214	0.0003	0.0131	0.0171	0.0114	0.0060

**Table 9:** Detailed results of SHAP values based on the best-case scenario and model for HOMA-IR index prediction.

Variables	Mean SHAP Value	Normalized Importance (%)
Insulin Level	0.386127	69.601879
Glucose Level	0.157894	28.461377
HbA1c	0.003909	0.704665
BMI	0.002708	0.488223
PLT	0.000789	0.142182
Glucose Lowering Therapy	0.000663	0.119595
LDL	0.000562	0.101317
Alive or Dead	0.000540	0.097258
HDL	0.000486	0.087628
Sum of 7 Other Variables	<0.000100	<0.200000

**Table 10:** Detailed results of SHAP values based on the first scenario and model for METS_IR index prediction.

Variables	Mean SHAP Value	Normalized Importance (%)
BMI	0.111903	52.295951
HDL	0.053610	25.053882
Triglyceride	0.030071	14.053200
Glucose Level	0.011547	5.396311
DM Medication Use	0.001301	0.607823
Hb1Ac	0.001093	0.510613
Disease Duration	0.000777	0.363008
Beta Blocker	0.000678	0.316647
LDL	0.000604	0.282355
Sum of 21 Other Variables	<0.002397	<1.01

**Table 11:** Detailed results of SHAP values based on the second scenario and model for METS_IR index prediction.

Variables	Mean SHAP Value	Normalized Importance (%)
BMI	0.188768	57.184101
Triglyceride	0.067544	20.461354
HDL	0.045996	13.933619
Glucose Level	0.010555	3.197529
Hb1Ac	0.002530	0.766396
GFR	0.001675	0.507397
Creatine	0.001600	0.484827
ESR	0.001590	0.481623
ULT Use	0.001551	0.469840
Sum of 21 Other Variables	<0.009	<2.51

**Table 12:** Detailed results of SHAP values based on the best-case scenario and model for TyG index prediction.

Variables	Mean SHAP Value	Normalized Importance (%)
Triglyceride	0.045210	70.606239
Glucose Level	0.017186	26.839294
Hb1Ac	0.000523	0.816865
Uric Acid	0.000251	0.392666
HDL	0.000200	0.311719
ALT	0.000157	0.244759
DM Medication Use	0.000132	0.206271
Creatine	0.000128	0.200562
Haemoglobin	0.000103	0.161071
Sum of 5 Other Variables	<0.0001	<0.220500

**Table 13:** Comparative analysis of the research findings and the existing literature.

Study	Prediction	Best Model	Performance Results
[16]	HOMA-IR	CATBOOST	78.02% Accuracy
	METS-IR	TabKANet	75.10% Accuracy
	TyG	CATBOOST	90.98% Accuracy
[17]	HOMA-IR	XGB (for 99 features)	86.80% Accuracy
	HOMA-IR	XGB (for 99 features)	87.70% Accuracy
	HOMA-IR	ANN (for 9 features)	86.20% Accuracy
[18]	Dataset A	XGB	0.64 MSE, 0.61 MAE, 0.85 $R^{2}$
	Dataset B (METS-IR)	XGB	0.61 MSE, 0.61 MAE, 0.87 $R^{2}$
	Dataset C (HOMA-IR)	XGB	0.79 MSE, 0.69 MAE, 0.83 $R^{2}$
[19]	HOMA-IR	SVM	87.00% Sensitivity, 93.00% Specificity
[20]	HOMA-IR	XGB	78.00% Accuracy
[21]	HOMA-IR	XGB	81.00% Accuracy
	HOMA-IR	Traditional Biostatistical Methods	70.20% Sensitivity, 52.70% Specificity
	TyG Index	Traditional Biostatistical Methods	52.70% Sensitivity, 78.30% Specificity
This Study	HOMA-IR	CATBOOST (Scenario 4)	0.9968 $R^{2}$, 0.0182 MAE, 0.9540 $D^{2}$
	METS-IR	CATBOOST (Scenario 4)	0.9856 $R^{2}$, 0.0136 MAE, 0.8958 $D^{2}$
	TyG Index	CATBOOST (Scenario 4)	0.9979 $R^{2}$, 0.0019 MAE, 0.9600 $D^{2}$

## Data Availability

The data are sensitive and belong to people applying to the Rheumatology Department of Recep Tayyip Erdoğan University Rize Training and Research Hospital.

## Abbreviations

The following abbreviations are used in this manuscript:

ACE	Angiotensin-Converting Enzyme Inhibitors
ADABOOST	Adaptive Boosting
ALT	Alanine Aminotransferase
ARB	Angiotensin II Receptor Blockers
AST	Aspartate Aminotransferase
BMI	Body Mass Index
BPH	Benign Prostatic Hyperplasia
CAD	Coronary Artery Disease
CATBOOST	Category Boosting
CCBs	Calcium Channel Blockers
CE	Cerebrovascular Event
CHF	Chronic Health Failure
CRP	C-Reactive Protein
ESR	Erythrocyte Sedimentation Rate
GA	Genetic Algorithm
GBM	Gradient Boosting Machine
GFR	Glomerular Filtration Rate
Hba1c	Glycosylated Haemoglobin
HDL	High-Density Lipoprotein
HOMA IR	Homeostatic Model Assessment of Insulin Resistance
LDL	Low-Density Lipoprotein
LGBM	Light Gradient Boosting Machine
MAE	Mean Absolute Error
MAPE	Mean Absolute Percentage Error
MCV	Mean Corpuscular Volume
MedAE	Median Absolute Error
METS IR	Metabolic Score for Insulin Resistance
MSE	Mean Squared Error
NSAID	Non-Steroidal Anti-Inflammatory Drug
PAD	Peripheral Artery Disease
PLT	Platelet
RMSE	Root Mean Squared Error
ROC	Receiver Operating Characteristics
SHAP	Shapley Additive Operations
TyG Index	Triglyceride-Glucose Index
ULT	Urate-Lowering Therapy
WBC	White Blood Cell
XGB	Extreme Gradient Boosting
$D^{2}$	The Square of Distance
$R^{2}$	The Square of the Correlation Coefficient

## Appendix A

**Table A1:** Hyperparameter search spaces for all models.

Model	Hyperparameter Name	Range
Extreme Gradient Boosting	N Estimators	Integer (100, 1000)
	Max Depth	Integer (2, 10)
	Learning Rate	Float (0.01, 0.2, log = *True*)
	Subsample	Float (0.5, 1.0)
	Col Sample By Tree	Float (0.5, 1.0)
	Col Sample By Level	Float (0.5, 1.0)
	Col Sample By Node	Float (0.5, 1.0)
	Min Child Weight	Float (1 × 10^−2^, 10, log = *True*)
	Gamma	Float (1 × 10^−8^, 5.0, log = *True*)
	Reg Alpha	Float (1 × 10^−8^, 10.0, log = *True*)
	Reg Lambda	Float (1 × 10^−8^, 10.0, log = *True*)
	Max Delta Step	Integer (0, 10)
	Objective	Reg: Squared Error
	Tree Method	Hist
Gradient Boosting Machine	N Estimators	Integer (100, 1000)
	Max Depth	Integer (3, 9)
	Learning Rate	Float (0.01, 0.2, log = *True*)
	Subsample	Float (0.5, 1.0)
	Max Features	Float (0.5, 1.0)
	Min Samples Split	Integer (2, 20)
	Min Samples Leaf	Integer (2, 20)
	Loss	Categorical [‘Squared Error’, ‘Absolute Error‘, ‘Huber’]
	Criterion	Categorical [‘Friedmann MSE’, ‘Squared Error’]
	Min Impurity Decrease	Float (0.0, 0.5)
Categorical Boosting	Iterations	Integer (100, 1000)
	Depth	Integer (3, 9)
	Learning Rate	Float (0.01, 0.2, log = *True*)
	Subsample	Float (0.5, 1.0)
	L2 Leaf Reg	Float (1 × 10^−8^, 1.0, log = *True*)
	RSM	Float (0.5, 1.0)
	Random Strength	Float (1 × 10^−8^, 1.0, log = *True*)
	Loss Function	Categorical [‘RMSE’, ‘MAE’, ‘Quantile’, ‘Poisson’]
	Min Data in Leaf	Integer (2, 10)
	Bagging Temperature	Float (0.0, 10.0)
	Border Count	Integer (32, 255)
	Grow Policy	Categorical [‘Symmetric Tree’, ‘Depthwise, ‘Lossguide]
Adaptive Boosting	Base Estimator	Categorical [‘Decision Tree’, ‘Extra Tree’, ‘Ridge’]
	N Estimator	Integer (100, 1000)
	Learning Tate	Float (0.01, 0.2, log = *True*)
	Max Depth	Integer (3, 9)
	Min Samples Split	Integer (3, 9)
	Min Samples Leaf	Float (0.1, 10.0, log = *True*)
	Max Features	
	Splitter	
	Criterion	
	Ridge Alpha	
	Loss	Categorical [‘Linear’, ‘Square’, ‘Exponential’]
Light Gradient Boosting	N Estimator	Integer (100, 1000)
	Max Depth	Integer (3, 9)
	Learning Rate	Float (0.01, 0.2, log = *True*)
	Subsample	Float (0.5, 1.0)
	Col Sample By Tree	Float (0.5, 1.0)
	Min Split Gain	Float (1 × 10^−8^, 1.0, log = *True*)
	Reg Alpha	Float (1 × 10^−8^, 1.0, log = *True*)
	Reg Lambda	Float (1 × 10^−8^, 1.0, log = *True*)
	Objective	Categorical [‘Regression’, ‘Regression L1’, ‘Quantile’]
	Num Leaves	Integer (16, 128)

## Appendix B

**Table A2:** Optimal hyperparameter configurations for XGB in HOMA-IR prediction.

Hyperparameter	Optimal Hyperparameter Values			
	Raw Data	Log-Transformed	GA + Raw Data	GA + Log-Transformed
N Estimators	231	643	998	842
Max Depth	2	2	2	8
Learning Rate	0.12622899788765707	0.03770908043939299	0.12891127130809157	0.013274574732055098
Subsample	0.6921907316206455	0.6119164692530034	0.960859181323126	0.5247589665008323
Col Sample By Tree	0.8928088726120675	0.9991826248009387	0.8552518431897034	0.9856981973410888
Col Sample By Level	0.9937392787762166	0.9727760585692848	0.7423937380639413	0.8726474992571234
Col Sample By Node	0.7782388924077213	0.730210577911097	0.9703686456816172	0.9861936096677629
Min Child Weight	1.6019834110455677	0.016809632984543178	0.02870029311136882	1.242074931126579
Gamma	8.556887599369384	0.0006025892470008067	0.0003722450944387166	4.983842999872669-05
Reg Alpha	2.661877152876055	0.04273684594798332	0.0001500771485838924	0.002291313301307255
Reg Lambda	4.380635969442609	0.10339934458470473	4.671219611416119	0.003389662028155613
Max Delta Step	8	6	10	5
Optimization Time	32.41 s	29.26 s	51.37 s	58.07 s

**Table A3:** Optimal hyperparameter configurations for XGB in METS-IR prediction.

Hyperparameter	Optimal Hyperparameter Values			
	Raw Data	Log-Transformed	GA + Raw Data	GA + Log-Transformed
N Estimators	565	803	875	601
Max Depth	2	2	2	2
Learning Rate	0.07210878031377675	0.1244776364211423	0.043790270942120346	0.07351270800094652
Subsample	0.7226505306886235	0.7933204207938301	0.6678674817065435	0.7335945438467372
Col Sample By Tree	0.7434742244681959	0.8895388581160938	0.7324097077853808	0.9793187965091078
Col Sample By Level	0.9261767674570135	0.8222686758326192	0.9452766829646932	0.7558428864030619
Col Sample By Node	0.9862958004605669	0.9767734647517989	0.9814483374915918	0.9287445212419853
Min Child Weight	0.051261704127667944	0.09620211354709783	0.3350267451135588	0.9710462634638231
Gamma	0.026604167190781784	0.0018712215292560383	2.507943635668113	1.7873104939481408
Reg Alpha	0.0001519981798077706	0.01414946792990126	0.33728763514228066	4.542016598311843
Reg Lambda	9.675896737387614	0.1478978247102793	0.0003174895529580056	0.0005728745084241472
Max Delta Step	5	8	10	2
Optimization Time	77.13 s	42.46 s	58.00 s	39.70 s

**Table A4:** Optimal hyperparameter configurations for XGB in TyG prediction.

Hyperparameter	Optimal Hyperparameter Values			
	Raw Data	Log-Transformed	GA + Raw Data	GA + Log-Transformed
N Estimators	859	871	933	849
Max Depth	2	2	3	3
Learning Rate	0.017082372554728564	0.02045170002825048	0.04368009306756808	0.11418346163616457
Subsample	0.5650580126887552	0.5474170357779929	0.8053625276429138	0.6038602970761711
Col Sample By Tree	0.9716963233445247	0.9397139519789325	0.9505021555844257	0.9949653398513811
Col Sample By Level	0.9188407751161801	0.9544633351849785	0.8978832188083598	0.9337634707428721
Col Sample By Node	0.9132739165296331	0.9261229612717614	0.9350878414647266	0.772063822734442
Min Child Weight	0.10705656320219632	1.1419570132444687	0.012301715607175138	0.06610763782316431
Gamma	4.752535005538394	2.0395307848073286	2.81873158774429	1.2200542910566178
Reg Alpha	1.217613136754007	2.6806571120317267	5.443072360268635	0.0001699170498657329
Reg Lambda	1.134313005321755	1.6697117685102416	0.13518407787320952	1.4382222378461583
Max Delta Step	3	8	7	4
Optimization Time	87.34 s	60.90 s	55.92 s	34.25 s

**Table A5:** Optimal hyperparameter configurations for GBM in HOMA-IR prediction.

Hyperparameter	Optimal Hyperparameter Values			
	Raw Data	Log-Transformed	GA + Raw Data	GA + Log-Transformed
N Estimators	803	957	960	899
Max Depth	9	2	8	6
Learning Rate	0.05199695791579833	0.0327872015521714	0.01710424926665799	0.011578134720936877
Subsample	0.6223380119305372	0.6354749672951843	0.7616677123336819	0.6695960278018113
Max Features	0.8396143320515793	0.890588501148555	0.965015987887853	0.8324557387632746
Min Samples Split	15	18	16	18
Min Samples Leaf	2	2	2	2
Loss	Squared Error	Squared Error	Squared Error	Squared Error
Criterion	Friedmann MSE	Friedmann MSE	Friedmann MSE	Friedmann MSE
Min Impurity Decrease	0.01410510284965743	0.003764443018410555	0.030167050892735907	0.0001307470624361957
Optimization Time	291.44 s	227.00 s	190.28 s	103.65 s

**Table A6:** Optimal hyperparameter configurations for GBM in METS-IR prediction.

Hyperparameter	Optimal Hyperparameter Values			
	Raw Data	Log-Transformed	GA + Raw Data	GA + Log-Transformed
N Estimators	990	950	468	503
Max Depth	3	2	2	3
Learning Rate	0.03504527155044838	0.15497400311101106	0.06272425204331815	0.07772652149917496
Subsample	0.5042986640823152	0.9375302134641357	0.5005365610079712	0.5607131399092081
Max Features	0.7583091691282605	0.7562638534599614	0.8836756708422276	0.8256975559931924
Min Samples Split	15	18	3	16
Min Samples Leaf	2	3	3	4
Loss	Huber	Absolute Error	Huber	Absolute Error
Criterion	Friedman MSE	Friedman MSE	Friedman MSE	Squared Error
Min Impurity Decrease	0.03028034714353617	0.006652842802575753	0.025825830327604604	0.006487180111816124
Optimization Time	216.02 s	305.06 s	110.67 s	121.22 s

**Table A7:** Optimal hyperparameter configurations for GBM in TyG prediction.

Hyperparameter	Optimal Hyperparameter Values			
	Raw Data	Log-Transformed	GA + Raw Data	GA + Log-Transformed
N Estimators	952	807	728	917
Max Depth	9	8	2	6
Learning Rate	0.01565691652702913	0.018366593903581096	0.05301788518687859	0.012392521548546863
Subsample	0.8481297292325993	0.9273928478326325	0.5435163296879532	0.6120656797462346
Max Features	0.8125584591359434	0.8830737189890445	0.9853520717102693	0.9648373042721223
Min Samples Split	17	2	11	10
Min Samples Leaf	3	5	3	5
Loss	Absolute Error	Absolute Error	Absolute Error	Absolute Error
Criterion	Friedman MSE	Squared Error	Friedman MSE	Friedman MSE
Min Impurity Decrease	0.00247525378307708	0.006713320488573553	0.04330412142989985	0.010676969958423526
Optimization Time	693.00 s	693.69 s	180.21 s	334.03 s

**Table A8:** Optimal hyperparameter configurations for CATBOOST in HOMA-IR prediction.

Hyperparameter	Optimal Hyperparameter Values			
	Raw Data	Log-Transformed	GA + Raw Data	GA + Log-Transformed
N Estimators	994	631	464	664
Max Depth	3	10	4	4
Learning Rate	0.0333760226555551	0.01925987580406569	0.08647710812081469	0.022121826558344777
Subsample	0.8681445549324297	0.5873046101834957	0.9750236947762791	0.578244419427861
RSM	0.9661942288222677	0.8523837464845166	0.9513214950547594	0.8293838339170754
L2 Leaf Reg	0.010757625293586166	0.9920116831604565	0.0002128523629584812	0.0001416541249560037
Random Strength	0.037781180361568745	0.15628272429222195	3.485143023417756	2.3529812159714394
Min Data in Leaf	15	22	23	8
Bagging Temperature	0.35877544952237983	5.859619446939263	8.240264619168629	2.6177240390973227
Border Count	107	224	208	163
Grow Policy	Symmetric Tree	Loss Guide	Symmetric Tree	Symmetric Tree
Loss Function	RMSE	RMSE	MAE	RMSE
Optimization Time	230.47 s	336.82 s	79.74 s	53.91 s

**Table A9:** Optimal hyperparameter configurations for CATBOOST in METS-IR prediction.

Hyperparameter	Optimal Hyperparameter Values			
	Raw Data	Log-Transformed	GA + Raw Data	GA + Log-Transformed
N Estimators	594	914	723	511
Max Depth	7	9	2	6
Learning Rate	0.058672187366254853	0.02118751677954625	0.08245349035413396	0.11888358319359102
Subsample	0.9405499823375387	0.5015746675595603	0.9971709236809473	0.5235002300053001
RSM	0.6898711112478085	0.8582924661608132	0.9391296713868933	0.5570865753254699
L2 Leaf Reg	0.0002826315500303557	7.879145556349846	0.8807542266549365	0.0001263365392168242
Random Strength	0.029580969950642265	0.9720138505275246	0.030416068180030138	0.001079047591621964
Min Data in Leaf	55	45	27	55
Bagging Temperature	5.555226559762334	2.8052732118299377	7.636213092239725	5.187387579492271
Border Count	225	251	217	219
Grow Policy	Loss Guide	Loss Guide	Symmetric Tree	Loss Guide
Loss Function	Poisson	RMSE	Poisson	RMSE
Optimization Time	261.13 s	273.20 s	112.31 s	110.67

**Table A10:** Optimal hyperparameter configurations for CATBOOST in TyG prediction.

Hyperparameter	Optimal Hyperparameter Values			
	Raw Data	Log-Transformed	GA + Raw Data	GA + Log-Transformed
N Estimators	394	701	996	919
Max Depth	9	5	2	5
Learning Rate	0.037744096054782385	0.012685809797939717	0.0636425730256645	0.024693137159088815
Subsample	0.6937658236692826	0.5575978043571027	0.6203859222352986	0.5891681558844453
RSM	0.9560735070670048	0.9840642260754346	0.6865247640211856	0.8206489753386716
L2 Leaf Reg	0.011104985847845585	9.051652905381085	0.004176824896847382	0.0007294831075967253
Random Strength	0.33537083735925755	0.06009998845088723	0.023500188195874478	0.0001567830280111549
Min Data in Leaf	63	38	13	49
Bagging Temperature	1.707878463693075	3.231946764243765	7.9652563210624505	4.160993140885787
Border Count	163	195	219	143
Grow Policy	Loss Guide	Symmetric Tree	Symmetric Tree	Symmetric Tree
Loss Function	RMSE	RMSE	MAE	RMSE
Optimization Time	311.07 s	214.73 s	68.01 s	74.95 s

**Table A11:** Optimal hyperparameter configurations for ADABOOST in HOMA-IR prediction.

Hyperparameter	Optimal Hyperparameter Values			
	Raw Data	Log-Transformed	GA + Raw Data	GA + Log-Transformed
Base Estimator	Ridge	Extra Tree	Decision Tree	Decision Tree
N Estimators	112	515	156	765
Learning Rate	0.01003382718939605	0.040207275987618675	0.07104744578085195	0.07396636651774036
Max Depth	N/A ^1^	10	10	9
Min Samples Split	N/A ^1^	10	8	4
Min Samples Leaf	N/A ^1^	3	3	3
Max Features	N/A ^1^	0.8177850675462831	0.9733060688154784	0.9492173521660261
Splitter	N/A ^1^	Best	Random	Best
Criterion	N/A ^1^	Absolute Error	Squared Error	Friedmann MSE
Ridge Alpha	0.22825038335607054	N/A ^2^	N/A ^2^	N/A ^2^
Loss	Exponential	Exponential	Exponential	Linear
Optimization Time	1113.40 s	1018.36 s	198.58 s	283.39 s

^1^ Ridge does not have these parameters. ^2^ Decision Tree and Extra Tree models do not have these parameters.

**Table A12:** Optimal hyperparameter configurations for ADABOOST in METS-IR prediction.

Hyperparameter	Optimal Hyperparameter Values			
	Raw Data	Log-Transformed	GA + Raw Data	GA + Log-Transformed
Base Estimator	Decision Tree	Extra Tree	Decision Tree	Extra Tree
N Estimators	401	478	355	787
Learning Rate	0.030185390123334547	0.06665926620419167	0.14252829453673083	0.08239938648168359
Max Depth	10	10	10	10
Min Samples Split	12	20	10	13
Min Samples Leaf	5	2	1	1
Max Features	0.9769761439475166	0.9710958556176378	0.9807677677386049	0.9746665698534374
Splitter	Random	Random	Random	Random
Criterion	Absolute Error	Friedman MSE	Friedman MSE	Friedman MSE
Ridge Alpha	N/A ^1^	N/A ^1^	N/A ^1^	N/A ^1^
Loss	Exponential	Square	Square	Linear
Optimization Time	1177.13 s	362.34 s	762.66 s	287.31 s

^1^ Ridge does not have these parameters.

**Table A13:** Optimal hyperparameter configurations for ADABOOST in TyG prediction.

Hyperparameter	Optimal Hyperparameter Values			
	Raw Data	Log-Transformed	GA + Raw Data	GA + Log-Transformed
Base Estimator	Decision Tree	Decision Tree	Decision Tree	Extra Tree
N Estimators	890	425	591	546
Learning Rate	0.08402083586100574	0.08014451361211752	0.10216891575638112	0.16469031627363512
Max Depth	10	10	10	9
Min Samples Split	9	14	4	19
Min Samples Leaf	4	2	6	3
Max Features	0.9753122320916467	0.9499876573363614	0.9998598760683104	0.8698673291137591
Splitter	Best	Random	Random	Random
Criterion	Friedman MSE	Squared Error	Friedman MSE	Friedman MSE
Ridge Alpha	N/A ^1^	N/A ^1^	N/A ^1^	N/A ^1^
Loss	Linear	Linear	Exponential	Linear
Optimization Time	645.21 s	482.43 s	225.05 s	294.42 s

^1^ Ridge does not have these parameters.

**Table A14:** Optimal hyperparameter configurations for LGBM in HOMA-IR prediction.

Hyperparameter	Optimal Hyperparameter Values			
	Raw Data	Log-Transformed	GA + Raw Data	GA + Log-Transformed
N Estimators	528	794	851	665
Max Depth	2	2	2	10
Learning Rate	0.012269402075257757	0.024908940337961087	0.09165340059398981	0.16148400338853136
Subsample	0.5951975579285192	0.7273748188462457	0.544323041484653	0.6626833481429724
Subsample Freq	9	8	4	6
Colsample By Tree	0.9761931592733434	0.9362265395224378	0.51580841946264	0.9594757767162099
Min Split Gain	5.437372364740818	9.708555463306793	0.0002667258276213963	0.013827288624955365
Min Child Samples	7	5	5	5
Min Child Weight	0.34107714838106623	0.3814667798972042	0.016365673283117096	0.08143640660298751
Reg Alpha	0.0004647432978200749	2.278035115212578	1.782365159366474	9.192520186578164
Reg Lambda	0.10536787189292866	1.023697560016334	0.40448736739149455	7.4071589473122115
Objective	Regression	Regression	Regression	Regression
Max Bin	431	407	103	461
Num Leaves	65	129	63	27
Boosting Type	GBDT	GBDT	Dart	Dart
Optimization Time	25.03 s	30.76 s	36.91 s	54.18 s

**Table A15:** Optimal hyperparameter configurations for LGBM in METS-IR prediction.

Hyperparameter	Optimal Hyperparameter Values			
	Raw Data	Log-Transformed	GA + Raw Data	GA + Log-Transformed
N Estimators	430	808	701	306
Max Depth	4	2	2	8
Learning Rate	0.023523492535033188	0.07457480713105315	0.047987177747785346	0.07204265648197099
Subsample	0.7808468899841456	0.9751697918939076	0.6291000203739738	0.520030667062009
Subsample Freq	2	6	2	1
Colsample By Tree	0.9149581970292564	0.5284849285973051	0.7948866972198582	0.773856962941405
Min Split Gain	5.719138601678863	3.740494223688223	0.007216155012299557	0.003964768340793771
Min Child Samples	9	5	5	5
Min Child Weight	0.0012621354055363126	0.02533911623932803	0.0872349835877214	0.004832987776783665
Reg Alpha	0.0038210998507970913	0.0003287211014537239	0.27563430596275384	0.0018149602719953804
Reg Lambda	1.441599330187265	1.6531587066753966	1.26701663226442	2.3529578812820358
Objective	Regression	Regression	Regression	Regression
Max Bin	193	285	310	366
Num Leaves	120	200	254	84
Boosting Type	GBDT	GBDT	GBDT	GBDT
Optimization Time	29.84 s	24.40 s	15.70 s	11.50 s

**Table A16:** Optimal hyperparameter configurations for LGBM in TyG prediction.

Hyperparameter	Optimal Hyperparameter Values			
	Raw Data	Log-Transformed	GA + Raw Data	GA + Log-Transformed
N Estimators	752	320	288	712
Max Depth	8	2	2	0.0187418108936603
Learning Rate	0.07883442869656208	0.07189532193784723	0.07384686503599316	2
Subsample	0.5094413655560925	0.9224926319919375	0.5004886823838539	0.6249040960043726
Subsample Freq	3	7	4	2
Colsample By Tree	0.993280033730148	0.8930977319900562	0.7510444385764798	0.694951317302785
Min Split Gain	7.426658530590672	2.0881433651511976	4.112090516658499	3.740215853973672
Min Child Samples	5	7	5	7
Min Child Weight	0.00568133045417771	5.305383742824826	0.2557530327742528	0.06479627370602355
Reg Alpha	3.689165305624637	0.0001823045184389674	1.809588200125185	1.0493224076906998
Reg Lambda	8.256227269634081	5.1387841490932814	1.958398167619824	2.0928991524709055
Objective	Regression	Regression	Regression	Regression
Max Bin	480	324	244	451
Num Leaves	73	53	15	26
Boosting Type	GBDT	GBDT	GBDT	GBDT
Optimization Time	53.15 s	14.96 s	27.06 s	17.40 s
